# A general and highly regioselective synthesis approach to multi-functionalized organoimido derivatives of Polyoxometalates

**DOI:** 10.1038/srep24759

**Published:** 2016-04-25

**Authors:** Yichao Huang, Jiangwei Zhang, Jian Hao, Yongge Wei

**Affiliations:** 1 Analysis and Test Center, Beijing University of Chemical Technology, Beijing 100029, P. R. China; 2 Key Lab of Organic Optoelectronics & Molecular Engineering of Ministry of Education, Department of Chemistry, Tsinghua University, Beijing 100084, P. R. China; 3 State Key Laboratory of Natural and Biomimetic Drugs, Peking University, Beijing 100191, P. R. China

## Abstract

Organoimidoylization of Polyoxometalates (POMs) can dramatically modify the electronic structures of POMs and gives rise to novel “value-adding” properties of the POMs for promising material applications including photo-electronic transformation and catalysis. To date, the preparation of multi-functionalized organoimido derivatives of POMs is generally conducted under strict condition and is time-consuming with limited yields. Herein, a series of regioselective polyorganoimido derivatives of POMs, *ocatant*- [Mo_6_O_13_(NAr)_3_(*μ*_2_-NAr)_3_]^2**−**^ (Ar = phenyl (**1**), *p*-methoxyphenyl (**2**) and *p*-ethylphenyl (**3**)), were synthesized with high selectivity and in good yields *via* a general and highly regioselective synthesis method, called as the one-octant synthesis protocol. The reaction was monitored by ESI-MS and the as-prepared products were studied by ESI-MS, IR, UV-Vis, EA, ^1^HNMR, single crystal XRD analysis and DFT calculations. The one-octant synthesis protocol here may serve as an idea method to design novel nanoscale POM-based organic-inorganic multi-functional hybrids.

Polyoxometalates (POMs) are a special class of inorganic oxide anion clusters consisting of early transition metals (e.g. Mo, W and V etc.) in their highest oxidation state with a wide range of properties, such as photonic, electronic and magnetic properties, leading to a multitude of applications in catalysis^1^,^2^,^3^,^4^,^5^, medicine^6^,^7^,^8^,^9^ and materials sciences^10^,^11^,^12^. Covalently incorporating some functionalized organic moieties on the POMs’ surfaces in a reliable and predefined manner holds promise to make the structure versatility more accessible and the design of advanced functional materials more rational^13^,^14^. Recently, organoimidoylization of POMs has gained particular attentions, because it can dramatically modify the electronic structures of POMs and gives rise to novel redox properties of related organic-inorganic hybrid materials. This benefits from the delocalized organic p-π electrons in arylimido derivatives of POMs, which may extend their conjugation to inorganic POM framework, resulting in strong d–p π interactions^15^,^16^,^17^. Owing to the excellent pioneering researches conducted by Proust^18^,^19^, Maatta^20^,^21^,^22^, Errington^23^, namely reactions of phosphinimines, isocyanates and aromatic amines (Scheme S1 in SI), organoimido derivatives of POMs can now be prepared with various imido-releasing reagents. It is worth mentioning that Strong and Maatta *et al.*^20^,^21^,^22^ has creatively isolated the desired higher-substituted derivatives from a mixture containing lower-substituted byproducts through one pot reaction of aryl isocyanates as the imido-releasing reagents. However, only the terminal O_t_ atoms in [Mo_6_O_19_]^2−^ could be replaced by arylimido ligands (Fig. 1a–c). Moreover, the synthetic condition in strictly anhydrous pyridine at high temperature for even several weeks have prevented the broad application of Maatta’s protocol. To date, polysubstituted organoimido derivatives of POMs, especially with bridging oxo group (O_b_) replacement mode are still rare, leaving a field that is full of challenges. Therefore, It should be conquered to further develop some reliable chemistry of polysubstituted organoimido derivatives of POMs.

The controllable preparation of mono- and disubstituted organoimido derivatives of [Mo_6_O_19_]^2−^ with high selectivity has also been achieved by the powerful DCC-dehydrating protocol in Peng and Wei’s groups^24^,^25^,^26^,^27^,^28^,^29^,^30^,^31^,^32^,^33^. Following this protocol, Ruhlmann and Wei *et al.* found that the bridging oxo group (O_b_) between two terminal organoimido groups can be replaced in the tri- and penta-substituted organoimido derivatives of [Mo_6_O_19_]^2−^ (Fig. 1e,g)^34^,^35^. But these products could only be obatined in approximately 30% yield and only one O_b_ atom could be substituted. This encouraged us to explore an effective route to obtain the desired polysubstituted organoimido derivatives of POMs with more O_b_ atom being substituted so as to construct building blocks for multi-functionalized POM-based materials. Herein, we report a highly regioselective one-octant synthesis protocol to directly synthesize the polysubstituted organoimido derivatives of hexamolybdates with the most congested replacement mode. The reaction system was monitored by ESI-MS and the as-prepared products were studied by single crystal XRD analysis, IR, UV-Vis, EA, ^1^HNMR as well as ESI-MS.

## Results and Discussions

### ESI-MS monitoring to optimize reaction conditions

**Figure 1:**
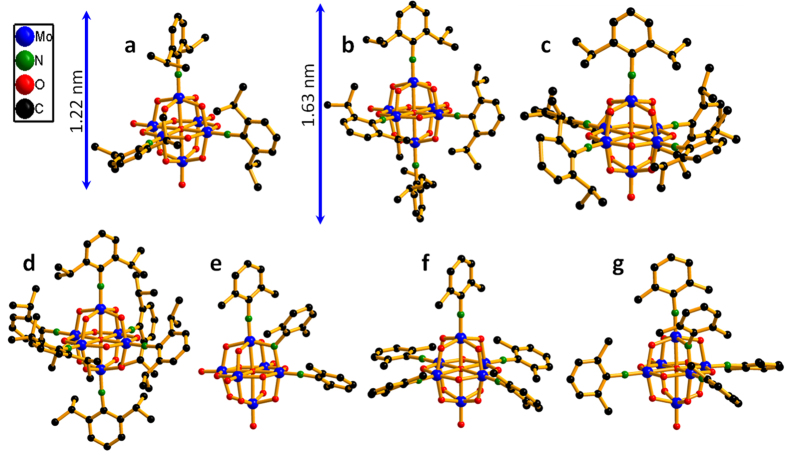
The ball and stick structures and sizes of some important polysubstituted derivatives of hexamolybdates. (**a**) [Mo_6_O_16_(NAr)_3_]^2−^ (**3-Ar**); (**b**) [Mo_6_O_15_(NAr)_4_]^2−^ (**4-Ar**); (**c**) [Mo_6_O_14_(NAr)_5_]^2−^ (**5-Ar**); (**d**) [Mo_6_O_13_(NAr)_6_]^2−^ (**6-Ar**); (**e**) [Mo_6_O_16_(NAr′)_2_(*μ*_2_-NAr′)]^2−^ (**3-Ar-III**); (**f**) [Mo_6_O_14_(NAr′)_5_]^2−^ (**5-Ar-I**); (**g**) [Mo_6_O_14_(NAr′)_4_(*μ*_2_-NAr′)]^2^ (**5-Ar-II**)^19^,^20^,^21^,^22^,^34^.

**Figure 2:**
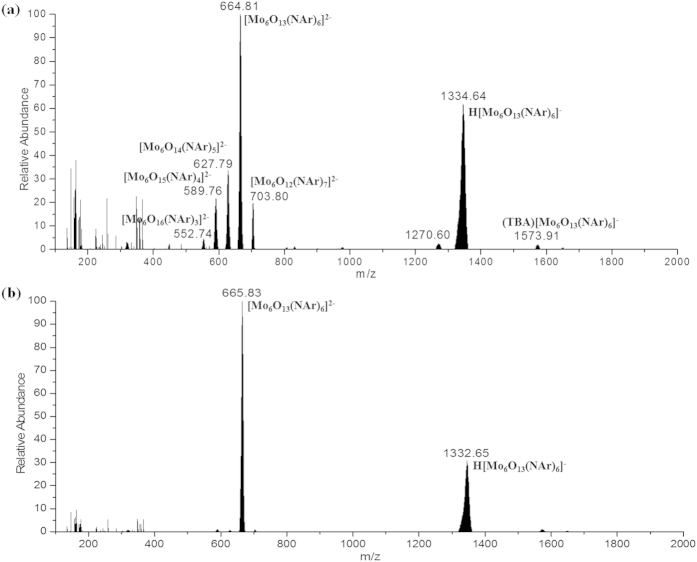
ESI mass spectrometry of the product in reaction (**a**) without L1HCl and reaction (**b**) with moderate L1HCl. For reaction (**a**), 1.62 g (TBA)_4_[Mo_8_O_26_] (0.75 mmol), 1.68 g aniline (18 mmol) and 4.13 g DCC (20 mmol) were dissolved in 30 mL anhydrous MeCN, then the solution was refluxing at 80 °C. After 12 h, 2 mL of the reaction solution was filtrated to remove the white precipitates of 1,3-dicyclohexylurea (DCU) and a black clear solution was obtained, and then the filtrate was poured into ether, resulting in precipitation, which was dissolved in 1 mL acetonitrile to obtain the ESI mass spectrometry, while for reaction (**b**), 1.49 g aniline (16 mmol) and 0.26 g L1HCl (2 mmol) was added into the reaction system instead.

**Figure 3:**
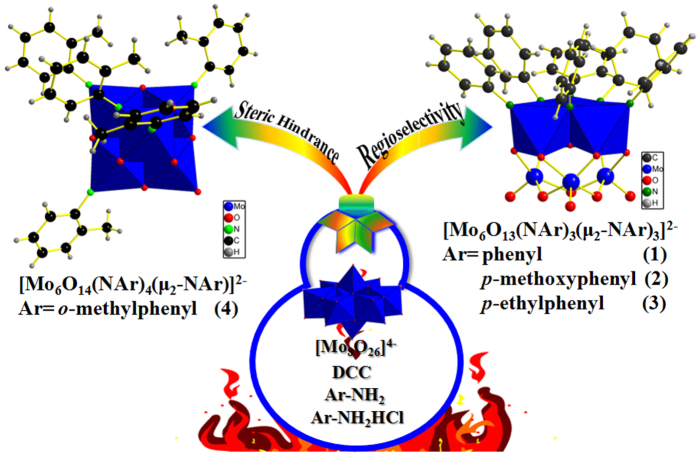
The highly regioselective synthesis procedure for polysubstituted organoimido derivatives of hexamolybdates.

**Figure 4:**
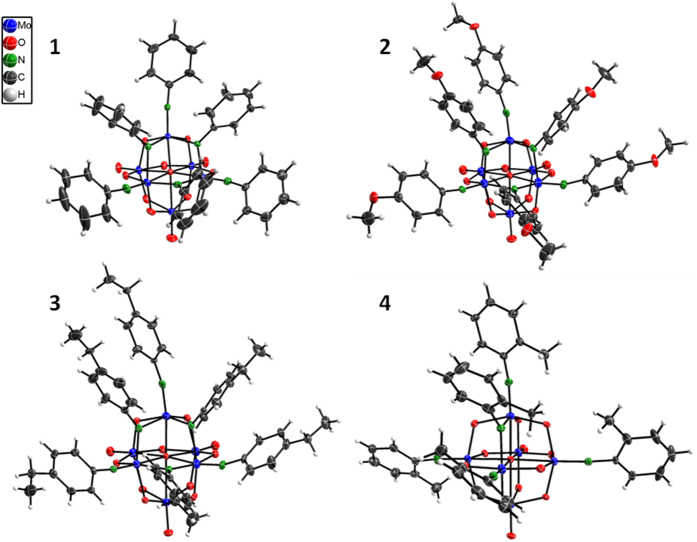
ORTEP drawings of cluster anions of compounds 1–4. Compounds 1 (top left), 2 (top right), 3 (bottom left) and 4 (bottom right). Thermal ellipsoids are drawn at the 30% probability level. Crystal data and structure refinement see SI.

As shown in Fig. 2, only very few examples of the polyorganoimido derivatives of lindqvist-POM have been reported and the fully substituted derivatives have not been obtained to date. Therefore, we first attempted to obtain full substituted organoimido derivatives by refluxing [Mo_8_O_26_]^4−^, aniline (L1), and DCC in a ratio of 0.75:18:20 following the DCC-dehydrating protocol^29^. The reaction process was monitored by mass spectrometry. Out of expectation, there were no peaks in ESI-MS matched the desired full substituted organoimido derivatives [Mo_6_O(NAr)_18_]^2−^. Interestingly, there was a major peak with 100% intensity located at 664.81 which belonged to a hexasubstituted organoimido derivative of hexamolybdates, [Mo_6_O_13_(NAr)_6_]^2−^. However, there were still some peaks located at 552.74 (3.74%), 589.76 (20.86%), 627.79 (32.53%), and 703.80 (17.01%) which belonged to tri-, tetra-, penta- and hepta-substituted derivatives of [Mo_6_O_19_]^2−^, respectively (Fig. 2a), which indicated that hexa-substituted derivative was the main product of the reaction but there were still some byproducts in this reaction. Our attempts to isolate the single crystal of such hexasubstituted derivative were in vain. Then [Mo_6_O_19_]^2−^ was selected to replace [Mo_8_O_26_]^4−^ as the reactant. Unfortunately, some mixtures of different substituted derivatives were obtained again. In consideration of the reaction can be speeded up and the product distribution can be affected by adding aromatic amine hydrochloride in our previous discovery^36^, aniline hydrochloride (L1HCl) was added to the reaction mixture to screen the best condition. By adjusting the ratio of anline and aniline hydrochloride, an optimized reaction condition was reached and shown in Fig. 3 (for synthesis details see Experimental section). From ESI-MS monitoring, the peaks matched well with the desired hexasubstituted organoimido derivative of hexamolybdate as shown in Fig. 2b. After recrystallization, the desired product was obtained as black crystals. Interestingly, from single crystal XRD analysis, an unprecedented regioselective one-octant hexasubstituted mode was discovered. Compared with the reported terminal hexasubstituted organoimido derivative of hexamolybdates^21^, *terminal*-[Mo_6_O_13_(NAr)_6_]^2−^ (**6-Ar**) (Fig. 1d), three O_t_ and three O_b_ groups in an octant of [Mo_6_O_19_]^2−^ were substituted by six aniline ligands respectively in this compound *ocatant*-[Mo_6_O_13_(NAr)_3_(*μ*_2_-NAr)_3_]^2−^ (Ar = phenyl (**1**)) (see Fig. 3 and ORTEP drawing of compound **1** in Fig. 4).

### Structure flexibility of the most congested replacement mode

It is obvious that the replacement mode of compound **1** is the most congested, while that of compound **6-Ar** is the roomiest one among all the isomers of hexasubstituted derivatives of hexamolybdates. Considering compound **1** and **6-Ar** are stable enough to be obtained under suitable conditions, we assumed that the most congested replacement mode in compound **1** can be extended to other aromatic amine ligands. In order to verify such a hypothesis, *p*-anisidine (L2) and *p*-ethylaniline (L3) were chosen to conduct the similar synthetic procedure, respectively. As we expected, another two similar products, *ocatant*- [Mo_6_O_13_(NAr)_3_(*μ*_2_-NAr)_3_]^2−^ (Ar = *p*-methoxyphenyl (**2**) and *p*-ethylphenyl (**3**)), were obtained (see Fig. 3 and ORTEP drawings of compounds **2** and **3** in Fig. 4). From compounds **2** and **3**, the same replacement mode was obviously observed again. It should be noted that the contribution of aromatic amine hydrochloride to the reaction is essential to obtain such octant hexasubstituted organoimido derivatives of hexamolybdate with high selectivity and purity. From compounds **1**–**3**, the obvious flexibilities and stabilities of the congested replacement mode in the octant hexasubstituted derivatives of hexamolybdates were definitely confirmed.

However, while a steric hindrance ligand, *o*-toluidine (L4), was exploited in the similar reaction. Another polysubstituted derivative of hexamolybdates, {Mo_6_O_14_[NC_6_H_4_(*o*-CH_3_)]_4_[*μ*_2_-NC_6_H_4_(*o*-CH_3_)]}^2−^ (**4**), was obtained as the mainly isolated products (see Fig. 4). In fact, compound **4** has the same replacement mode as the previously reported pentasubstituted derivative, [Mo_6_O_14_(2,6-Me_2_-NC_6_H_3_)_4_(*μ*_2_-2,6-Me_2_-NC_6_H_3_)]^2−^ (Fig. 1g, (5**-Ar-II**))^34^, reported by Hao and Ruhlmann *et al.* It may due to the similar steric hindrance in L4 and 2,6-dimethylaniline. However, if the steric hindrance becomes stronger, only terminal hexasubstituted organoimido derivative of hexamolybdates, *terminal*-[Mo_6_O_13_(NAr)_6_]^2−^ (Fig. 1d, 6**-Ar**), could be obtained, even though a 20-fold molar excess of 2,6-diisopropylphenyl isocyanate were added into the reaction of [Mo_6_O_19_]^2−^ in refluxing pyridine for three weeks^21^. These results show that the steric hindrance can obviously affect the formation of replacement modes and the steric hindrance factor may serve as an option to obtain products with special replacement modes in some extent.

### Structure characterizations

The structures of compounds **1**–**3** have been confirmed definitively by single crystal X-ray diffraction analysis. All of these compounds reveal a preference for mutual *cis*-coordination of [NAr] ligands, suggesting that the presence of extant [NAr] groups exert an activating effect at proximal O_b_ atoms. These compounds have two different types of nitrogen atoms including three N_t_ (terminal N) atoms and three N_b_ (bridging *μ*_2_-N) atoms, which have different hybridization patterns. Take compound **1** for example, the short Mo-N_t_ bond lengths range from 1.747 to 1.756 Å and near-linear Mo-N_t_-C bond angles range from 168.3 to 172.5°, which are typical of Mo≡N triple bond character where N_t_ atoms possess sp hybridization^15^. While Mo-N_b_ bond lengths range from 1.943 to 2.082 Å and Mo-N_b_-C bond angles range from 123.7 to 130.3° which is consistent with that the bridging N_b_ atoms possess sp^2^ hybridization mode^34^. Furthermore, the N_b_ atom is coplanar with the other three atoms where it bonds. But the dihedral angle between the benzene ring bonded to N_b_ and the Mo-N_b_-Mo plane ranges from 60.765 to 76.412°, which is deviated far from 0°. This suggests that the non-hybridized p orbitals of the N_b_ atoms are not conjugated with the benzene ring of the bridging ligands. This situation would result in the reduction of conjugative interaction between the bridging imido ligand and the hexamolybdate cluster. Correspondingly, the bond length from the imido-bearing Mo atom to O_c_ atom was significantly shorter than the other Mo–O_c_ due to the enhanced “*trans* influence”^16^. Selected bond lengths and bond angles of compounds **1**–**4** are listed in Table S1–S3.

**Figure 5:**
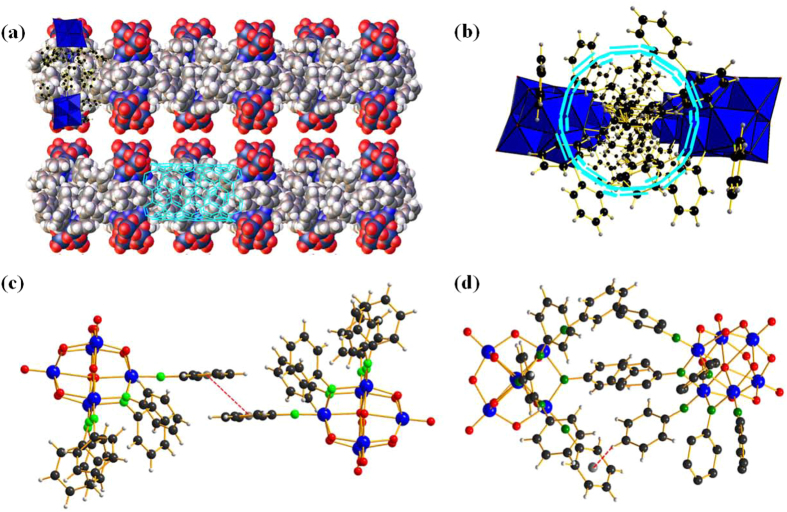
Crystal structures of compound 1. (**a**) Packing along *c* axis (drawn by space filling). (**b**) Packing along *b* axis (drawn by polyhedron as well as ball and stick). (**c**) The weak π…π stacking interactions between the phenyl rings of the adjacent molecules. (**d**) The intermolecular C-H…π interactions in the crystal structure.

The packing modes of compound **1** are projected in the direction along *b* and *c* axis, respectively (Fig. 5a,b). The cluster anions pack one by one to form dimeric units through π-π stacking with the same remote aromatic ring orientated in the opposite direction, then further assemble into layer-by-layer structures based on 1D infinite chain substructures. As shown in Fig. 5c, the phenyl rings of the adjacent molecules are parallel to each other and the closest distance between the C atoms of such two parallel phenyl rings is *ca* 0.380 nm, showing the existence of π…π stacking interactions. Furthermore, the π-stacking structure also involves intermolecular C-H…π interactions, with C-H/π distances of 0.247 ~ 0.317 nm and angles of 156° ~ 168° (Fig. 5d). In addition, the solvent-accessible voids in the crystals can form nanosized carbon-channels which are very similar to the carbon nanotubes (CNT).

### Spectra characterizations

**Figure 6:**
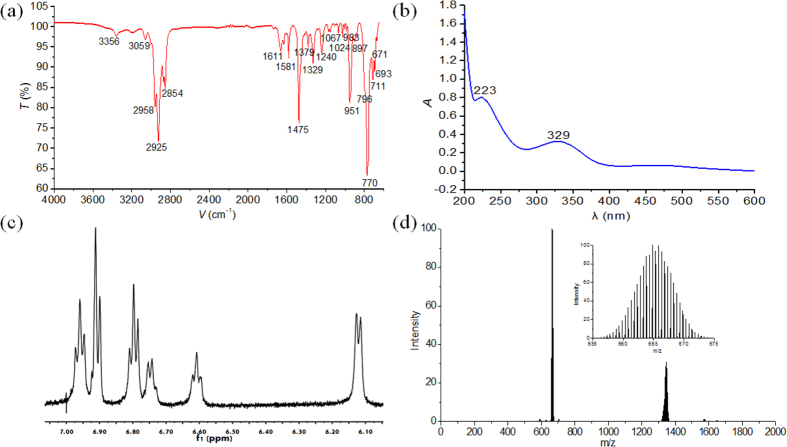
The spectra and ESI-MS characterizations of compound 1. (**a**) IR, (**b**) UV-Vis, (**c**) ^1^H NMR spectra and (**d**) ESI-MS.

The IR spectra of compounds **1–4** show special Mo-O and Mo-N stretching bands in the region from 650 to 1000 cm^−1^. The weak and medium bands ranging from 995 cm^−1^ to 972 cm^−1^ can be assigned for terminal Mo ≡ N bonds of the polysubstituted arylimido derivatives of a hexamolybdate^33^,^34^. The bridging Mo-N-Mo asymmetric stretching vibrations display from 850 cm^−1^ to 900 cm^−1^ as a medium band in compounds **1**–**4**. The most distinctive feature in IR spectra of these compounds **1**–**4** is that they all present similar characteristics in the Mo-O stretching region of 1000–700 cm^−1^ (Fig. S1 in SI). Take compound **1** as example (Fig. 6), the peaks located at 951, cm^−1^ are ascribed to the stretching vibration of Mo = O_t_ groups. The asymmetric Mo-O-Mo stretching vibration is found near 796 and 749 cm^−1^ for compounds **1**. UV–Vis absorption spectra of compounds **1**–**4** were measured so as to investigate the electronic properties. Compared with the lowest-energy electronic transition absorption band of parent [Mo_6_O_19_]^2−^ locating around 325 nm, compounds **1–4** show obviously bathochromic shift to 329 nm, 347, 332 and 352 nm, respectively, due to the strongly enhancement of conjugation when O_t_ atoms were substituted by organoimido ligands NAr. The increasing number of organoimido ligands substituted O_t_ atoms in parent [Mo_6_O_19_]^2−^ cluster will make such bathochromic shift become more obvious. This is in excellent consistent with the above X-ray structure study (Fig. 6 and Fig. S2 in SI). ^1^H NMR spectra of these compounds show clearly resolved signals. The integration matches well with their structures. Take compound **1** for example, compared with the corresponding ^1^H NMR of aniline ligand, the signals in the ^1^H NMR spectra of compound **1** exhibit upfield shift owing to the stronger electron-donating nature of organoimido ligands than oxo groups in POMs cluster. Compared to terminal ligands, the aromatic protons on bridging imido ligands show further upfield resonance signals. As the X-ray structure discussion demonstrates that the Mo-N_t_Ar conjugation is much stronger than Mo-(N_b_Ar)-Mo conjugation. Thus aromatic hydrogen atoms in phenyl ring of N_t_Ar shows higher chemical shifts than that of phenyl ring of N_b_Ar. Another contrary effect is that the diamagnetic anisotropy of these two kinds of phenyl rings also has effect on partial offset downfield shift of phenyl ring of N_b_Ar (Fig. 6 and Fig. S3 in SI). Compounds **1–4** have also been investigated by ESI-MS, whose picks matched well with the desired products (Fig. 6 and Fig. S4 in SI).

### Investigating the steric hindrance effect by DFT calculations

**Figure 7:**
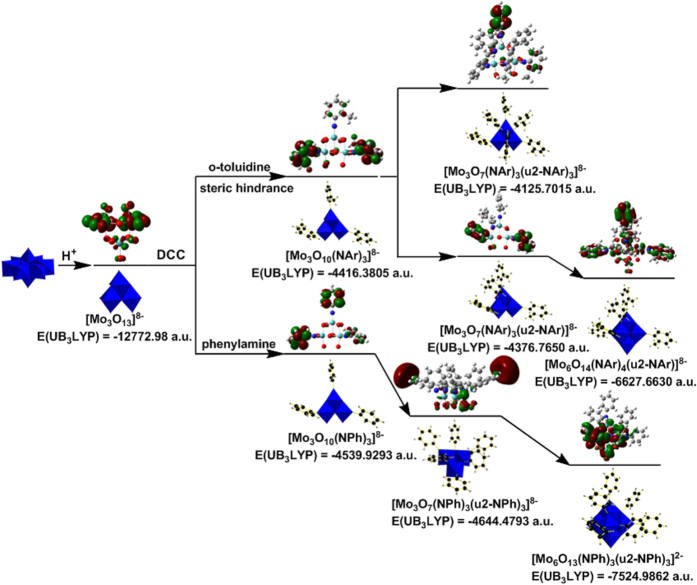
Study of the steric hindrance effect to the forming of polysubstituted deriviatives of hexamolybdates based on DFT calculations. The energy E_(UB3LYB)_, HUMO and LUMO Molecular Orbital(MO) of compound 1 and 4 and the related intermediates are calculated by DFT calculations.

The steric hindrance effect to the forming of these regioselective polysubstituted derivatives of hexamolybdates could be comprehended with the relevant DFT calculations as the followings (Fig. 7). Since hydrolysis reaction of [Mo_8_O_26_]^4−^ will going and hydrolyzes into small building blocks, like [Mo_2_O_7_]^2−^ and [Mo_3_O_13_]^8−^37, through a degradation and reassembly process. We may take [Mo_3_O_13_]^8−^ as the model to investigate the steric hindrance effect. Therefore, under the given condition, [Mo_3_O_10_(NAr)_3_]^8−^ will be formed *via* DCC dehydration process. DFT results show that the steric hindrance factor obviously affected the formation of the final products. If steric hindrance did not exist, the intact fragment, [Mo_3_O_7_(NAr)_3_(*μ*_2_-NAr)_3_]^8−^, would be formed because of the generation of 3c π bonds (Mo-N_b_-Mo)^38^ and such a fragment possess lower energy and was more stable than {Mo_3_O_7_[NC_6_H_4_(*o*-CH_3_)]_6_}^8−^. Then it will be further stabilized to form Lindqvist-POM structure by later joining fragment [Mo_3_O_6_]^6+^. The energy of compounds **1**–**3** were reduced dramatically and these compounds became stable enough to be isolated as single crystals. However, if the steric hindrance group existed, the formation of {Mo_3_O_7_[NC_6_H_4_(*o*-CH_3_)]_3_[*μ*_2_-NC_6_H_4_(*o*-CH_3_)]_3_}^8−^ could be much more difficult than [Mo_3_O_7_(NAr)_3_(*μ*_2_-NAr)_3_]^8−^, forming another different precursor, which could also be further stabilized by joining fragment [Mo_3_O_6_]^6+^ and reacted with the remaining aromatic amine ligands forming compound **4**.

## Conclusions

**Figure 8:**
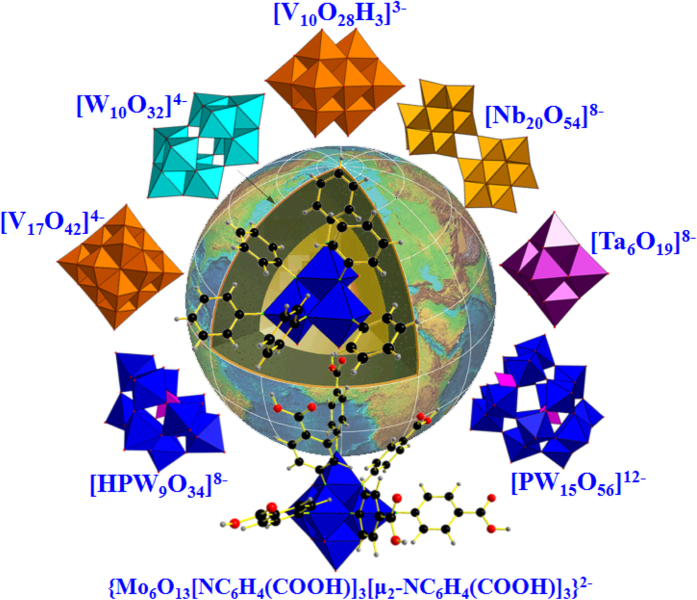
The related investigations are under study in our laboratory.

In summary, three unprecedented regioselective one-octant hexasubstituted derivatives of hexamolybdates with the most congested replacement mode have been designed and synthesized in a controllable way with high selectivity and good yields *via* the one-octant synthesis protocol. The most crowded replacement mode in compounds **1**–**3** is flexible and stable enough to be extended to a class of aromatic amine ligands. Furthermore, the steric hindrance factor obviously affects the formation of structure-directing precursors which can control the formation of special replacement modes. The steric hindrance effect to the forming of these products was investigated by DFT calculations. All in all, the highly regioselective one-octant synthesis protocol here may serve as an reasonable method to design specific multi-functionalized organic-inorganic hybrid materials with high selectivity and good yields. The related investigations are under way in our laboratory as follows (Fig. 8):

1. Applying other POM structure units to capture the [Mo_3_O_7_(NAr)_3_(*μ*_2_-NAr)_3_]^8−^ fragment, forming novel mixed-metal organic functionalized hybrid materials;
2. Extending to other organic amines with remote functionality (such as carboxyl group) to generate multi-carboxyl clusters.

## Methods

### Materials and characterizations

All syntheses and manipulations were performed under N_2_ gas, all other chemicals, including solvents, were commercially available as reagent grade from Adamas-beta^®^. (TBA)_2_Mo_6_O_19_ and (TBA)_4_Mo_8_O_26_, were synthesized according to literature methods^39^ and dried before use. The aniline (L1), *p*-anisidine (L2), *p*-ethylaniline (L3), *o*-toluidine (L4) and their hydrochlorides were also dried before use. Acetonitrile was dried by refluxing in the presence of CaH_2_ and was distilled prior to use. *N,N′*-Dicyclohexylcarbodiimide (DCC) was used directly without further purification. IR spectra were measured by using KBr pellets and recorded on a Perkin Elmer FT-IR spectrometer. UV-Vis spectra were measured in acetonitrile with UV2100s spectrophotometer. The mass spectra were obtained by using an ion trap mass spectrometer (Thermofisher LTQ), negative mode was chosen for the experiments (capillary voltage 33 V), and sample solution (in acetonitrile) was infused into the ESI source at a flow rate of 300 μL min^**−**1^. Elemental analyses were performed by Elementar Analysensysteme GmbH (vario EL). ^1^H NMR spectra were obtained on a JEOL JNM-ECA400 spectrometer and reported in ppm.

### X-ray crystallography

Suitable single crystals were selected. Data collections were performed by graphite-monochromated Mo-Kα radiation (λ = 0.71073 Å). Data reduction, cell refinement and experimental absorption correction were performed with the software package of Rigaku RAPID AUTO (Rigaku, 1998, ver 2.30). The structures were solved by direct methods and refined against F^2^ by full-matrix least-squares. All non-hydrogen atoms were refined anisotropically. All calculations were carried out by the program package of SHELXTL ver 5.1^40^,^41^ and Olex2 ver 1.2.6^42^.

### Synthesis of (TBA)_2_[Mo_6_O_13_(NC_6_H_5_)_3_(*μ*_2_-NC_6_H_5_)_3_], compound 1

1.62 g (TBA)_4_Mo_8_O_26_ (0.75 mmol), 1.49 g aniline (16 mmol), 0.26 g aniline hydrochloride (2 mmol) and 4.13 g DCC (20 mmol) were dissolved in 30 mL anhydrous MeCN, then the solution was refluxing at 80 °C for 12 h. Then the reaction solution was filtrated to remove the white precipitates of 1,3-dicyclohexylurea (DCU) and a black clear solution was obtained, and then the filtrate was poured into ether, resulting in precipitation. After the solution became clear, the supernatant liquid was poured off. The title compounds could be obtained as black powdered products (73% yields based on Mo). ^1^H NMR (400 MHz, CDCl_3_, ppm): δ = 0.94 (t, 24H, TBA-H), 1.48 (sextet, 16H, TBA-H), 1.60 (quintet, 16H, TBA-H), 3.33 (t, 16H, TBA-H), 6.12 (d, 6H, Ar-H), 6.62 (t, 3H, Ar-H), 6.75 (d, 3H, Ar-H), 6.82 (t, 6H, Ar-H), 6.92 (d, 6H, Ar-H), 6.97 (t, 6H, Ar-H). IR (KBr pellet, major absorbances, cm^−1^): 3356, 2958, 2925, 2854, 1611, 1581, 1475, 1379, 1329, 1240, 1067, 1024, 983, 951, 897, 796, 770, 711, 693, 671. UV-Vis (MeCN, nm): λ_1_ = 223, λ_2_ = 329. ESI mass spectrometry (MeCN): calcd m/z = 1572.77, (TBA)[Mo_6_O_13_(NC_6_H_5_)_6_]^−^; 1331.3, [HMo_6_O_13_(NC_6_H_5_)_6_]^−^; 665.15, [Mo_6_O_13_(NC_6_H_5_)_6_]^2−^; found 1572.93, 1331.11, 664.98, respectively. Elemental analysis (calcd., found for C_68_H_102_Mo_6_N_8_O_13_): H (5.63, 5.66), C (46.02, 45.99), N(6.15, 6.17).

### Synthesis of (TBA)_2_{Mo_6_O_13_[NC_6_H_4_(*p*-OCH_3_)]_3_[*μ*_2_-NC_6_H_4_(*p*-OCH_3_)]_3_}, compound 2

1.62 g (TBA)_4_Mo_8_O_26_ (0.75 mmol), 1.97 g *p*-methoxyaniline (16 mmol), 0.32 g *p*-methoxyaniline hydrochloride (2 mmol) and 4.13 g DCC (20 mmol) were dissolved in 30 mL anhydrous MeCN, then the solution was refluxing at 80 °C for 12 h. Then the reaction solution was filtrated to remove the white precipitates of DCU and a black clear solution was obtained, and then the filtrate was poured into ether, resulting in precipitation. After the solution became clear, the supernatant liquid was poured off. The title compounds could be obtained as black powdered products (75% yields based on Mo). ^1^H NMR (400 MHz, CDCl_3_, ppm): δ = 0.95 (t, 24H, TBA-H), 1.48 (sextet, 16H, TBA-H), 1.51 (quintet, 16H, TBA-H), 1.62 (s, 18H, CH_3_-O-Ar), 3.35 (t, 16H, TBA-H), 6.11/6.63 (d, 12H, Ar-H), 6.82/6.93 (d, 12H, Ar-H). IR (KBr pellet, major absorbances, cm^−1^): 3434, 2960, 2933, 2873, 1627, 1578, 1479, 1382, 1345, 1318, 1281, 1249, 1165, 1126, 1024, 972, 943, 921, 900, 845, 795, 739, 665, 614. UV-Vis (MeCN, nm): λ_1_ = 237, λ_2_ = 347. ESI mass spectrometry (MeCN): calcd m/z = 1752.93, (TBA) {Mo_6_O_13_[NC_6_H_4_(*p*-OCH_3_)]_6_}^−^; 1511.46, {HMo_6_O_13_[NC_6_H_4_(*p*-OCH_3_)]_6_}^−^; 755.23, {Mo_6_O_13_[NC_6_H_4_(*p*-OCH_3_)]_6_}^2−^; found: 1753.06, 1511.66, 755.33. Elemental analysis (calcd., found for C_74_H_114_Mo_6_N_8_O_19_): H(5.80, 5.76), C (44.51, 44.54), N (5.64, 5.62).

### Synthesis of (TBA)_2_{Mo_6_O_13_[NC_6_H_4_(*p*-C_2_H_5_)]_3_[*μ*_2_-NC_6_H_4_(*p*-C_2_H_5_)]_3_}, compound 3

1.62 g (TBA)_4_Mo_8_O_26_ (0.75 mmol), 1.95 g 4-ethylaniline (16 mmol), 0.32 g 4-ethylaniline hydrochloride (2 mmol) and 4.13 g DCC (20 mmol) were dissolved in 30 mL anhydrous MeCN, then the solution was refluxing at 80 °C for 12 h. Then the reaction solution was filtrated to remove the white precipitates of DCU and a black clear solution was obtained, and then the filtrate was poured into ether, resulting in precipitation. After the solution became clear, the supernatant liquid was poured off. The title compounds could be obtained as black powdered products (69% yields based on Mo). ^1^H NMR (400 MHz, CDCl_3_, ppm): δ = 0.95 (t, 24H, TBA-H), 1.48 (sextet, 16H, TBA-H), 1.50 (quintet, 16H, TBA-H), 1.52/2.48 (t, 18H/12H, CH_3_-CH_2_-Ar), 3.33 (t, 16H, TBA-H), 6.04 (d, 6H, Ar-H), 6.60 (d, 6H, Ar-H), 6.79 (d, 12H, Ar-H). IR (KBr pellet, major absorbances, cm^−1^): 3358, 2960, 2927, 2874, 1660, 1632, 1491, 1380, 1329, 1237, 1171, 1067, 1026, 995, 961, 944, 916, 887, 793, 766, 726, 698, 668. UV-Vis (MeCN, nm): λ_0_ = 221, λ_1_ = 262, λ_2_ = 332. ESI mass spectrometry (MeCN): calcd m/z = 1747.14, (TBA){Mo_6_O_13_[NC_6_H_4_(*p*-C_2_H_5_)]_6_}^−^; 1505.67, {HMo_6_O_13_[NC_6_H_4_(*p*-C_2_H_5_)]_6_}^−^; 752.33, {Mo_6_O_13_[NC_6_H_4_(*p*-C_2_H_5_)]_6_}^2−^; found: 1747.25, 1505.98, 751.17. Elemental analysis (calcd., found for C_74_H_114_Mo_6_N_8_O_19_): H(6.72, 6.69), C (48.33, 48.29), N (5.59, 5.63).

### Synthesis of (TBA)_2_{Mo_6_O_14_[NC_6_H_4_(*o*-CH_3_)]_4_[*μ*_2_-NC_6_H_4_(*o*-CH_3_)]}, compound 4

1.62 g (TBA)_2_Mo_8_O_26_ (0.75 mmol), 1.71g *o*-toluidine (16 mmol), 0.29 g *o*-toluidine hydrochloride (2 mmol) and 4.13 g DCC (20 mmol) were dissolved in 30 mL anhydrous MeCN, then the solution was refluxing at 80 °C for 12 h. Then the reaction solution was filtrated to remove the white precipitates of DCU and a black clear solution was obtained, and then the filtrate was poured into ether, resulting in precipitation. After the solution became clear, the supernatant liquid was poured off. The title compounds could be obtained as black powdered products (50% yields based on Mo). ^1^H NMR (400 MHz, CDCl_3_, ppm): δ = 0.91 (t, 24H, TBA-H), 1.41 (sextet, 16H, TBA-H), 1.57 (quintet, 16H, TBA-H), 2.17–2.73 (m, 18H, CH_3_-Ar), 3.26 (t, 16H, TBA-H), 6.20 (d, 1H, Ar-H), 6.34 (d × d, 2H, Ar-H), 6.45 (d × d, 2H, Ar-H), 6.72 (d × d × d, 4H, Ar-H), 6.82 (d × d × d, 3H, Ar-H), 6.85 (d × d × d, 5H, Ar-H), 7.06 (q × d × d, 4H, Ar-H), 7.17 (q × d × d, 3H, Ar-H). IR (KBr pellet, major absorbances, cm^**−**1^): 3057, 2960, 2933, 2873, 1636, 1589, 1473, 1456, 1380, 1328, 1239, 1193, 1152, 1117, 1041, 995, 940, 914, 883, 749, 710, 661. UV-Vis (MeCN, nm): λ_0_ = 222, λ_1_ = 268, λ_2_ = 352. ESI mass spectrometry (MeCN): calcd m/z = 1567.80, (TBA){Mo_6_O_14_[NC_6_H_4_(*o*-CH_3_)]_5_}^−^; 1326.33, {HMo_6_O_14_[NC_6_H_4_(*o*-CH_3_)]_5_}^−^; 662.66, {Mo_6_O_14_[NC_6_H_4_(*o*-CH_3_)]_5_}^2−^; fund: 1567.68, 1326.42, 662.83. Elemental analysis (calcd., found for C_74_H_114_Mo_6_N_8_O_19_): H(6.01, 5.96), C (44.49, 44.45), N (5.39, 5.42).

### Recrystallization of compounds 1–4

1.5 g compounds **1**–**4** were redissolved in 10 mL MeCN and transferred into a bottle with ether respectively. After slow gaseous diffusion and crystallization, compounds **1**–**4** were obtained as black crystalline products.

### DFT calculations

All of the calculations presented herein were carried out with Gaussian09 program package. The structures of each stationary point were fully optimized by using the B3LYP method, in combination with the LANL2DZ basis set for molybdenum and vanadium atoms and the 6–31 + G(d) basis set for main group elements. Configuration optimized before Mülliken charge analysis. The calculations were completed on the “Explorer 100” cluster system of Tsinghua National Laboratory for Information Science and Technology.

## Additional Information

**How to cite this article**: Huang, Y. *et al.* A general and highly regioselective synthesis approach to multi-functionalized organoimido derivatives of Polyoxometalates. *Sci. Rep.* **6**, 24759; doi: 10.1038/srep24759 (2016).

## Supplementary Material

Supplementary Information

## References

[b1] Yin Q. *et al.* A Fast Soluble Carbon-Free Molecular Water Oxidation Catalyst Based on Abundant Metals. Science 328, 342–345 (2010). 20223949 10.1126/science.1185372

[b2] Al-Oweini R. *et al.* Photocatalytic Water Oxidation by a Mixed-Valent Mn^III^_3_Mn^IV^O_3_ Manganese Oxo Core that Mimics the Natural Oxygen-Evolving Center. Angew. Chem. Int. Ed. 53, 11182–11185 (2014). 10.1002/anie.201404664 25066304

[b3] Rausch B., Symes M. D., Chisholm G. & Cronin L. Decoupled catalytic hydrogen evolution from a molecular metal oxide redox mediator in water splitting. Science 345, 1326–1330 (2014). 25214625 10.1126/science.1257443

[b4] Shi D. *et al.* Merging of the photocatalysis and copper catalysis in metal-organic frameworks for oxidative C-C bond formation. Chem. Sci. 6, 1035–1042 (2015). 10.1039/c4sc02362e PMC5811127 29560191

[b5] Li Q., Wei Y., Hao J., Zhu Y. & Wang L. Unexpected CC Bond Formation via Doubly Dehydrogenative Coupling of Two Saturated sp3 C−H Bonds Activated with a Polymolybdate. J. Am. Chem. Soc. 129, 5810–5811 (2007). 17429975 10.1021/ja070600z

[b6] Rhule J. T., Hill C. L., Judd D. A. & Schinazi R. F. Polyoxometalates in Medicine. Chem. Rev. 98, 327–358 (1998). 11851509 10.1021/cr960396q

[b7] Ogata A. *et al.* Antitumour effect of polyoxomolybdates: induction of apoptotic cell death and autophagy in *in vitro* and *in vivo* models. Brit. J. Cancer 98, 399–409 (2008). 18087283 10.1038/sj.bjc.6604133 PMC2361451

[b8] Yang H.-K. *et al.* Polyoxometalate–biomolecule conjugates: A new approach to create hybrid drugs for cancer therapeutics. Bioorg. Med. Chem. Lett. 23, 1462–1466 (2013). 23337597 10.1016/j.bmcl.2012.12.081

[b9] She S. *et al.* Aliphatic Organoimido Derivatives of Polyoxometalates Containing a Bioactive Ligand. Chem. –Eur. J. 20, 16987–16994 (2014). 25346326 10.1002/chem.201404317

[b10] Busche C. *et al.* Design and fabrication of memory devices based on nanoscale polyoxometalate clusters. Nature 515, 545–549 (2014). 25409147 10.1038/nature13951

[b11] Hu B. *et al.* Inorganic-organic hybrid polymer with multiple redox for high-density data storage. Chem. Sci. 5, 3404–3408 (2014).

[b12] Vasilopoulou M., Douvas A. M., Palilis L. C., Kennou S. & Argitis P. Old Metal Oxide Clusters in New Applications: Spontaneous Reduction of Keggin and Dawson Polyoxometalate Layers by a Metallic Electrode for Improving Efficiency in Organic Optoelectronics. J. Am. Chem. Soc. 137, 6844–6856 (2015). 25951374 10.1021/jacs.5b01889

[b13] Proust A. *et al.* Functionalization and post-functionalization: a step towards polyoxometalate-based materials. Chem. Soc. Rev. 41, 7605–7622 (2012). 22782306 10.1039/c2cs35119f

[b14] Dolbecq A., Dumas E., Mayer C. R. & Mialane P. Hybrid Organic−Inorganic Polyoxometalate Compounds: From Structural Diversity to Applications. Chem. Rev. 110, 6009–6048 (2010). 20666374 10.1021/cr1000578

[b15] Peng Z. Rational Synthesis of Covalently Bonded Organic–Inorganic Hybrids. Angew. Chem. Int. Ed. 43, 930–935 (2004). 10.1002/anie.200301682 14966875

[b16] Zhang J., Xiao F., Hao J. & Wei Y. The chemistry of organoimido derivatives of polyoxometalates. Dalton. Trans. 41, 3599–3615 (2012). 22358852 10.1039/c2dt11948j

[b17] Healey M. R., Best S. P., Goerigk L. & Ritchie C. A Heteroaromatically Functionalized Hexamolybdate. Inorganics 3, 82–100 (2015).

[b18] Proust A. *et al.* Phenylimido derivatives of [Mo_6_O_19_]^2−^: syntheses, X-ray structures, vibrational, electrochemical, 95Mo and ^14^N NMR studies. Inorg. Chim. Acta. 224, 81–95 (1994).

[b19] Gouzerh P. & Proust A. Main-Group Element, Organic, and Organometallic Derivatives of Polyoxometalates. Chem. Rev. (Washington, D C) 98, 77–111 (1998). 10.1021/cr960393d 11851500

[b20] Strong J. B. *et al.* A New Class of Functionalized Polyoxometalates: Synthetic, Structural, Spectroscopic, and Electrochemical Studies of Organoimido Derivatives of [Mo_6_O_19_]^2−^. J. Am. Chem. Soc. 122, 639–649 (2000).

[b21] Strong J. B., Haggerty B. S., Rheingold A. L. & Maatta E. A. A superoctahedral complex derived from a polyoxometalate: the hexakis(arylimido)hexamolybdate anion [Mo_6_(NAr)_6_O_13_H]^−^. Chem. Commun. (Cambridge), 1137–1138 (1997).

[b22] Strong J. B., Ostrander R., Rheingold A. L. & Maatta E. A. Ensheathing a Polyoxometalate: Convenient Systematic Introduction of Organoimido Ligands at Terminal Oxo Sites in [Mo_6_O_19_]^2−^. J. Am. Chem. Soc. 116, 3601–3602 (1994).

[b23] Clegg W. *et al.* Functionalization of [Mo_6_O_19_]^2−^ with aromatic amines: synthesis and structure of a hexamolybdate building block with linear difunctionality. J. Chem. Soc., Chem. Commun. 455–456 (1995).

[b24] Xu L. *et al.* Towards Main-Chain-Polyoxometalate-Containing Hybrid Polymers: A Highly Efficient Approach to Bifunctionalized Organoimido Derivatives of Hexamolybdates. Angew. Chem. Int. Ed. 41, 4129–4132 (2002). 10.1002/1521-3773(20021104)41:21<4129::AID-ANIE4129>3.0.CO;2-R 12412104

[b25] Xu B. *et al.* Synthesis and Optical Properties of Conjugated Polymers Containing Polyoxometalate Clusters as Side-Chain Pendants. Chem. Mater. 17, 2841–2851 (2005).

[b26] Lu M. *et al.* Hybrid Molecular Dumbbells: Bridging Polyoxometalate Clusters with an Organic π-Conjugated Rod. Angew. Chem. Int. Ed. 41, 1566–1568 (2002). 10.1002/1521-3773(20020503)41:9<1566::aid-anie1566>3.0.co;2-q 19750667

[b27] Kang J. *et al.* Molecular and Polymeric Hybrids Based on Covalently Linked Polyoxometalates and Transition-Metal Complexes. Angew. Chem. Int. Ed. 44, 6902–6905 (2005). 10.1002/anie.200501924 16206307

[b28] Yin P. *et al.* A Double-Tailed Fluorescent Surfactant with a Hexavanadate Cluster as the Head Group. Angew. Chem. Int. Ed. 50, 2521–2525 (2011). 10.1002/anie.201006144 21370329

[b29] Wei Y., Xu B., Barnes C. L. & Peng Z. An Efficient and Convenient Reaction Protocol to Organoimido Derivatives of Polyoxometalates. J. Am. Chem. Soc. 123, 4083–4084 (2001). 11457161 10.1021/ja004033q

[b30] Xiao F. *et al.* Polyoxometalatocyclophanes: Controlled Assembly of Polyoxometalate-Based Chiral Metallamacrocycles from Achiral Building Blocks. J. Am. Chem. Soc. 132, 5956–5957 (2010). 20380378 10.1021/ja101671q

[b31] Yin P. *et al.* Self-Recognition of Structurally Identical, Rod-Shaped Macroions with Different Central Metal Atoms during Their Assembly Process. J. Am. Chem. Soc. 135, 4529–4536 (2013). 23444907 10.1021/ja400656j

[b32] Zhang J. *et al.* Nanoscale Chiral Rod-like Molecular Triads Assembled from Achiral Polyoxometalates. J. Am. Chem. Soc. 132, 14–15 (2010). 20000667 10.1021/ja907535g

[b33] Zhu Y. *et al.* Bottom-Up Construction of POM-Based Macrostructures: Coordination Assembled Paddle-Wheel Macroclusters and Their Vesicle-like Supramolecular Aggregation in Solution. J. Am. Chem. Soc. 135, 17155–17160 (2013). 24111901 10.1021/ja408228b

[b34] Hao J. *et al.* Unprecedented Replacement of Bridging Oxygen Atoms in Polyoxometalates with Organic Imido Ligands. Angew. Chem. Int. Ed. 47, 2626–2630 (2008). 10.1002/anie.200704546 18297664

[b35] Hao J. *et al.* Unprecedented Organoimido-Derivatised Lacunary Polyoxometalates. Chem. -Eur. J. 18, 2503–2506 (2012). 22298376 10.1002/chem.201103830

[b36] Wu P. *et al.* An Easy Route to Monofunctionalized Organoimido Derivatives of the Lindqvist Hexamolybdate. Eur. J. Inorg. Chem. 2004, 2819–2822 (2004).

[b37] Zhang Y. *et al.* Two unprecedented aromatic guanidines supramolecular chains self-assembled by hydrogen bonding interaction. J. Mol. Struct. 1097, 145–150 (2015).

[b38] Yan L., Jin M., Song P. & Su Z. Electronic Properties of Unprecedented Bridging Organoimido-Substituted Hexamolybdate: New Insights from Density Functional Theory Study. J. Phys. Chem. B 114, 3754–3758 (2010). 20199037 10.1021/jp909336z

[b39] Filowitz M., Ho R. K. C., Klemperer W. G. & Shum W. Oxygen-17 nuclear magnetic resonance spectroscopy of polyoxometalates. 1. Sensitivity and resolution. Inorg. Chem. 18, 93–103 (1979).

[b40] Sheldrick G. M. A short history of SHELX. Acta. Crystallogr. A 64, 112–122 (2008). 18156677 10.1107/S0108767307043930

[b41] Huebschle C. B., Sheldrick G. M. & Dittrich B. ShelXle: a Qt graphical user interface for SHELXL. J. Appl. Crystallogr. 44, 1281–1284 (2011). 22477785 10.1107/S0021889811043202 PMC3246833

[b42] Dolomanov O. V. *et al.* OLEX2: a complete structure solution, refinement and analysis program. J. Appl. Crystallogr. 42, 339–341 (2009).

