# Supplementary material for: A general and highly regioselective synthesis approach to multi-functionalized organoimido derivatives of Polyoxometalates
Source: Sci Rep. 2016 Apr 25;6:24759. doi: 10.1038/srep24759 (PMC4842987; doi:10.1038/srep24759)
Supplement: Supplementary Information [file srep24759-s1.pdf]

Supporting materials

A general and highly regioselective synthesis approach to multi-functionalized organoimido derivatives of Polyoxometalates

Yichao Huang[1,2,3,+], Jiangwei Zhang[2,+], Jian Hao[1,*], Yongge Wei[2,3,*]

[1]Analysis and Test Center, Beijing University of Chemical Technology, Beijing 100029, P. R. China.

[2]Key Lab of Organic Optoelectronics & Molecular Engineering of Ministry of Education, Department of Chemistry, Tsinghua University, Beijing 100084, P. R. China.

[3]State Key Laboratory of Natural and Biomimetic Drugs, Peking University, Beijing 100191, P. R. China.

[+]These authors contributed equally to this publication.

Correspondence and requests for materials should be addressed to J.H and Y.G.W. (E-mail: xhaojian@163.com; E-mail: yonggewei@mail.tsinghua.edu.cn.)

Table of Contents

1. Crystallographic data 3

2. Schematic illustration of traditional synthetic procedures for organoimido derivatives..4

3. Tables of selected bond lengths for compounds 1-4 5

4. IR spectra 6

5. UV/Vis spectra...8

6. ^{1}H NMR data...11

7. ESI mass spectrometry of compounds 1-4...13

8. DFT calculations...16

9. References...36

1. Crystallographic data

Crystal data and structure refinement for compound **1**:

$(TBA)_2[Mo_6O_{13}(NC_6H_5)_3(\mu_2\text{-}NC_6H_5)_3]$, $C_{136}H_{204}Mo_{12}N_{16}O_{26}$, M_r = 3630.43, monoclinic, space group $P2_1/c$, a = 21.9635, b = 23.588, c = 29.5265 Å, α = γ = 90°, β = 92.881°,V = 15277.3 Å^3, Z = 4, T = 293 K, 30000 reflections measured, R_1(final) = 0.0437, wR_2 = 0.1028.

Crystal data and structure refinement for compound **2**:

$(TBA)_2\{Mo_6O_{13}[NC_6H_4(p\text{-}OCH_3)]_3[\mu_2\text{-}NC_6H_4(p\text{-}OCH_3)]_3\}.(Et)_2O$, $C_{78}H_{124}Mo_6N_8O_{20}$, M_r = 2069.50, monoclinic, space group $P2_1/c$, a = 24.731, b = 15.0180, c = 23.3337 Å, α = γ = 90°, β = 90.628°, V = 8665.9 Å^3, Z = 4, T = 100 K, 17004 reflections measured, R_1(final) = 0.0480, wR_2 = 0.1035.

Crystal data and structure refinement for compound **3**:

$(TBA)_2\{Mo_6O_{13}[NC_6H_4(p\text{-}C_2H_5)]_3[\mu_2\text{-}NC_6H_4(p\text{-}C_2H_5)]_3\}.(Et)_2O$, $C_{84}H_{142}Mo_6N_8O_{14}$, M_r = 2063.71, monoclinic, space group $P2_1/c$, a = 25.021, b = 14.8777, c = 23.5478 Å, α = γ = 90°, β = 91.106°, V = 8764.2 Å^3, Z = 4, T = 293 K, 17180 reflections measured, R_1(final) = 0.0449, wR_2 = 0.1278.

Crystal data and structure refinement for compound **4**:

$(TBA)_2\{Mo_6O_{14}[NC_6H_4(o\text{-}CH_3)]_4[\mu_2\text{-}NC_6H_4(o\text{-}CH_3)]\}$, $C_{67}H_{107}Mo_6N_7O_{14}$, M_r = 1810.27, monoclinic, space group $P2_1$, a = 15.103, b = 16.520, c = 17.245 Å, α = γ = 90°, β = 112.525°, V = 3974.4 Å^3, Z = 2, T = 109 K, 15627 reflections measured, R_1(final) = 0.0506, wR_2 = 0.1390.

The structures of compounds **1-4** have been deposited to CCDC with the entry number CCDC-1033546, 1050912, 1408703 and 1408704, which can be obtained free of charge from The Cambridge Crystallographic Data Centre *via* www.ccdc.cam.ac.uk/data_request/cif.

2. Schematic illustration of traditional synthetic procedures for organoimido derivatives

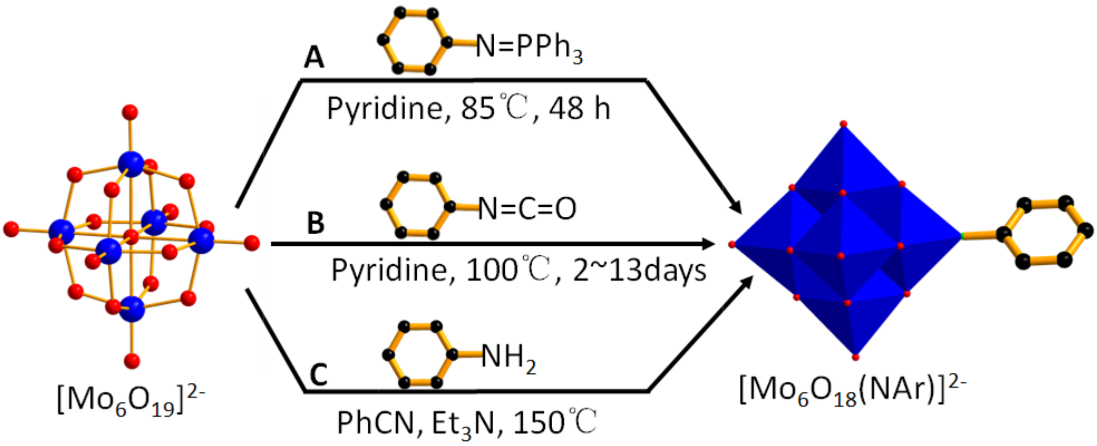

Scheme S1 Schematic illustration of traditional synthetic procedures for organoimido derivatives of polyoxometalates.[1-6] These synthesis protocols contain reactions of phosphinimines (A), isocyanates (B) and aromatic amines (C), which require strict condition in anhydrous pyridine at high temperature for a long time and subsequent tedious bench operations for purification and separation of the desired but low-yield products.

3. Tables of selected bond lengths and bond angles for compounds 1-4

Table S1 Selected bond lengths (Å) and bond angles (°) of compound 1.

Selected bond lengths (Å)				Selected bond angles (°)			
Mo1-N2	1.747	Mo2-N4	1.957	Mo1-N2-C7	171.90	Mo1-N4-C19	124.90
Mo2-N1	1.754	Mo2-N6	2.082	Mo2-N1-C1	168.30	Mo1-N5-C25	126.58
Mo3-N3	1.747	Mo3-N5	2.064	Mo3-N3-C13	172.50	Mo3-N5-C25	123.70
Mo1-N4	2.065	Mo3-N6	1.943	Mo2-N6-C31	125.90	Mo3-N6-C31	130.30
Mo1-N5	1.949			Mo2-N4-C19	125.90		

Table S2 Selected bond lengths (Å) of compounds 1-4.

Compounds	Bond Lengths (Å)					
**1**	Mo1-O1	2.164	Mo2-O1	2.172	M3-O1	2.174
	Mo4-O1	2.542	Mo5-O1	2.538	Mo6-O1	2.525
**2**	Mo1-O13	2.159	Mo2-O13	2.170	M3-O13	2.195
	Mo4-O13	2.531	Mo5-O13	2.536	Mo6-O13	2.522
**3**	Mo1-O13	2.146	Mo2-O13	2.197	M3-O13	2.208
	Mo4-O13	2.515	Mo5-O13	2.572	Mo6-O13	2.528
**4**	Mo1-O1	2.244	Mo2-O1	2.314	M3-O1	2.236
	Mo4-O1	2.272	Mo5-O1	2.448	Mo6-O1	2.477

Table S3 Selected bond lengths (Å) and bond angles (°) of compound 4.

Selected bond lengths (Å) of compound 4			
Mo1-N1	1.761	Mo2-N2	1.726
Mo3-N3	1.739	Mo4-N5	1.752
Mo3-N4	1.985		
**Selected angles (°) of compound 4**			
Mo1-N1-C1	169.173	Mo2-N2-C8	166.822
Mo3-N3-C15	175.681	Mo4-N5-C29	175.722
Mo1-N4-C22	121.581	Mo3-N4-C22	127.287

4. IR spectra

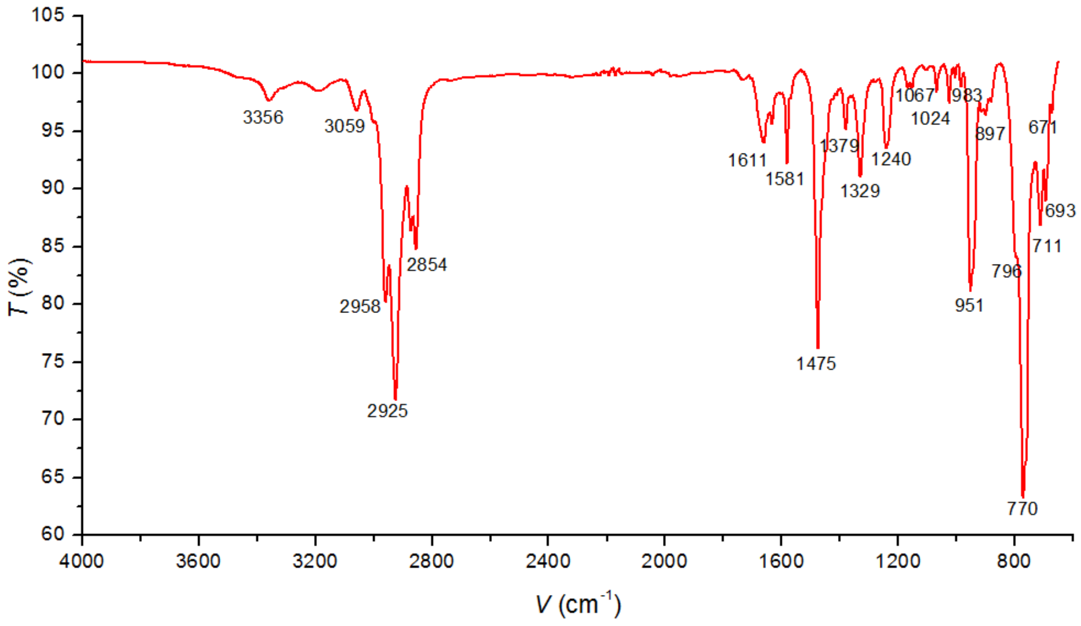

Fig. S1a The IR spectra of compound **1**.

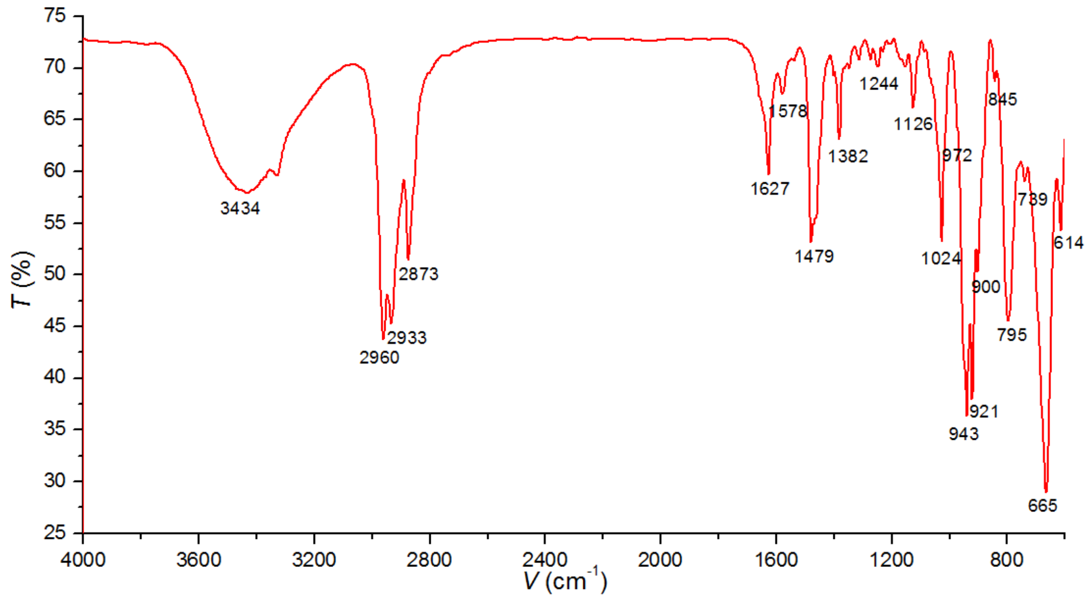

Fig. S1b The IR spectra of compound **2**.

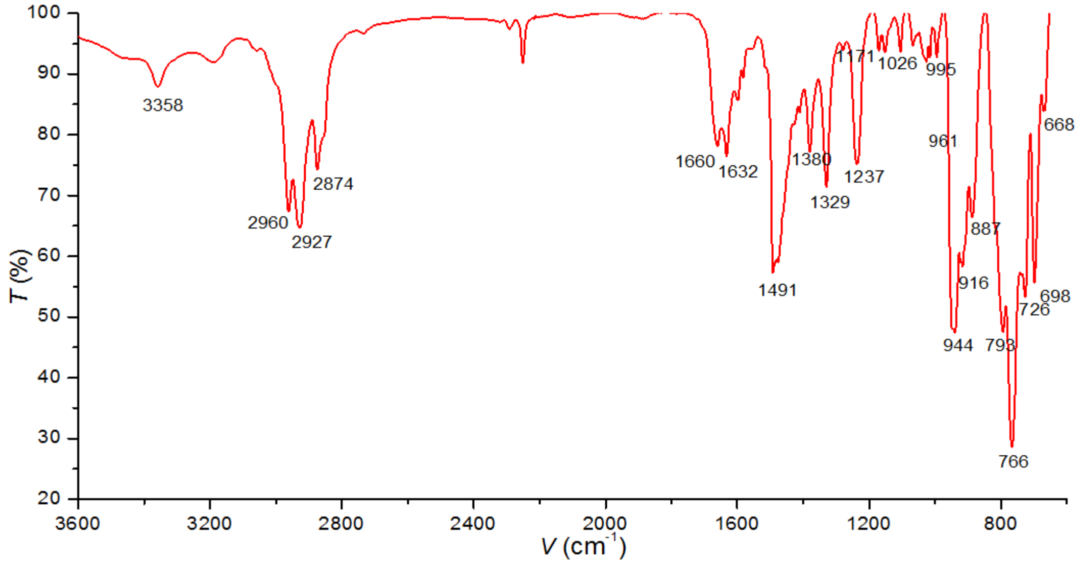

Fig. S1c The IR spectra of compound **3**.

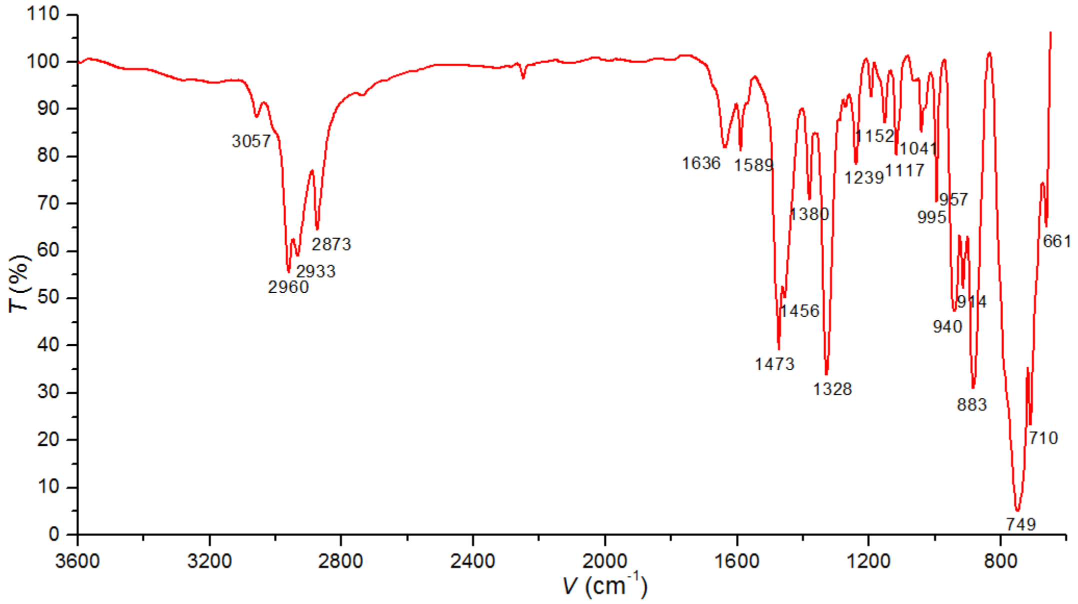

Fig. S1d The IR spectra of compound **4**.

5. UV/Vis spectra

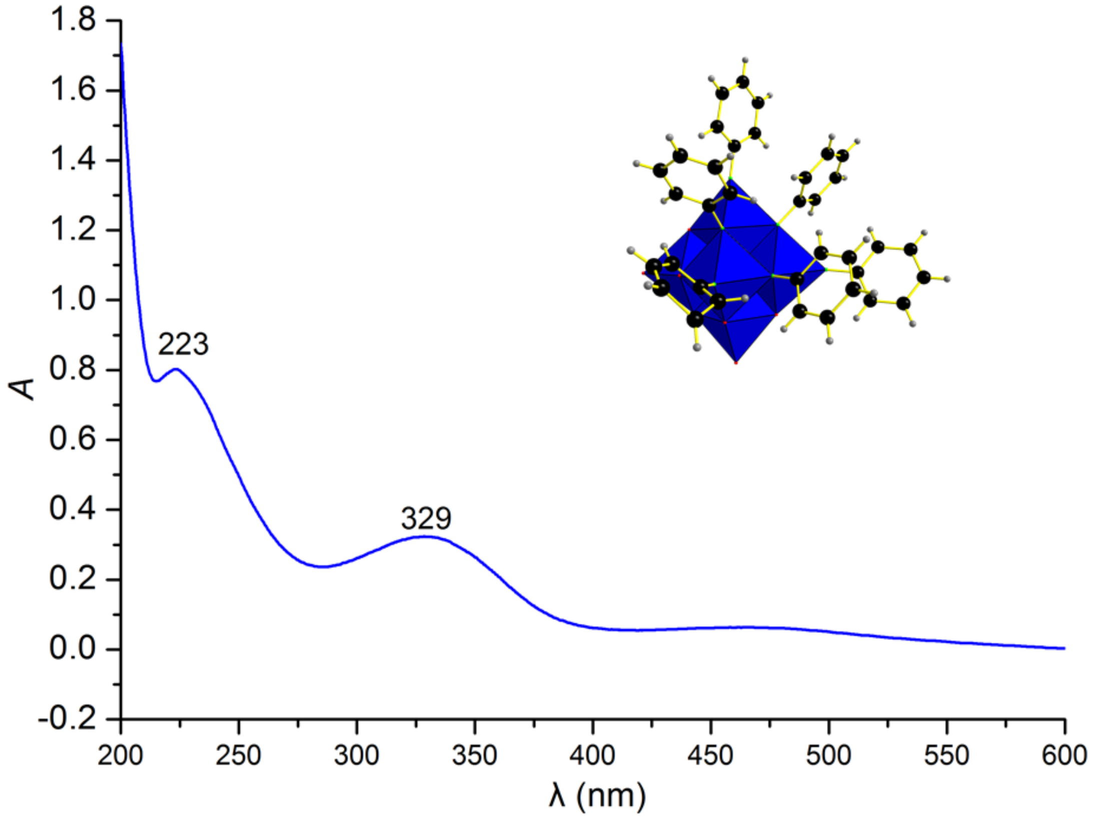

Fig. S2a UV/Vis spectra of compound **1**.

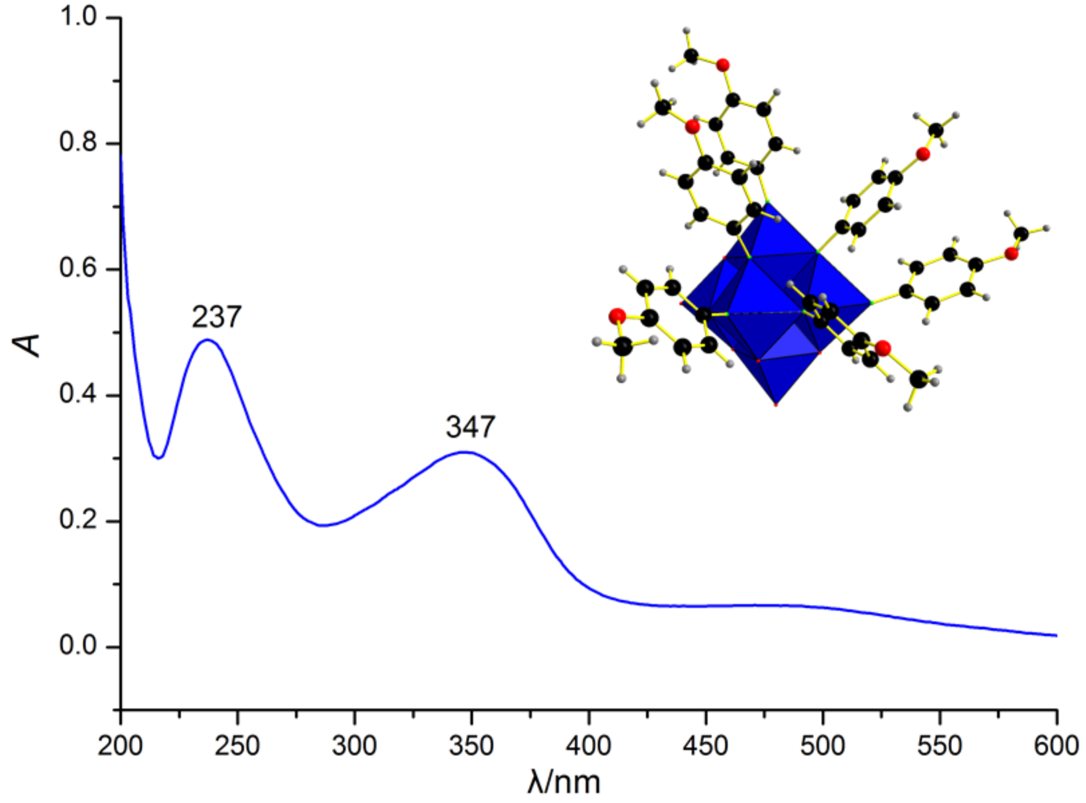

Fig. S2b UV/Vis spectra of compound **2**.

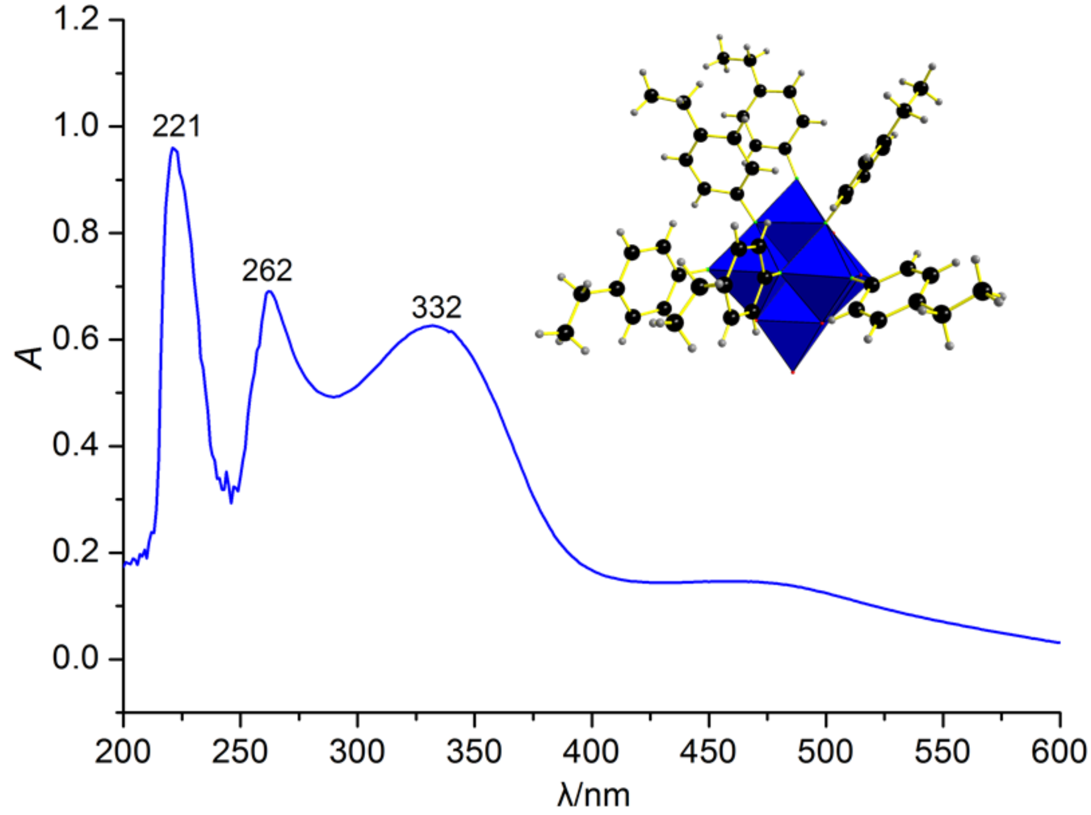

Fig. S2c UV/Vis spectra of compound **3**.

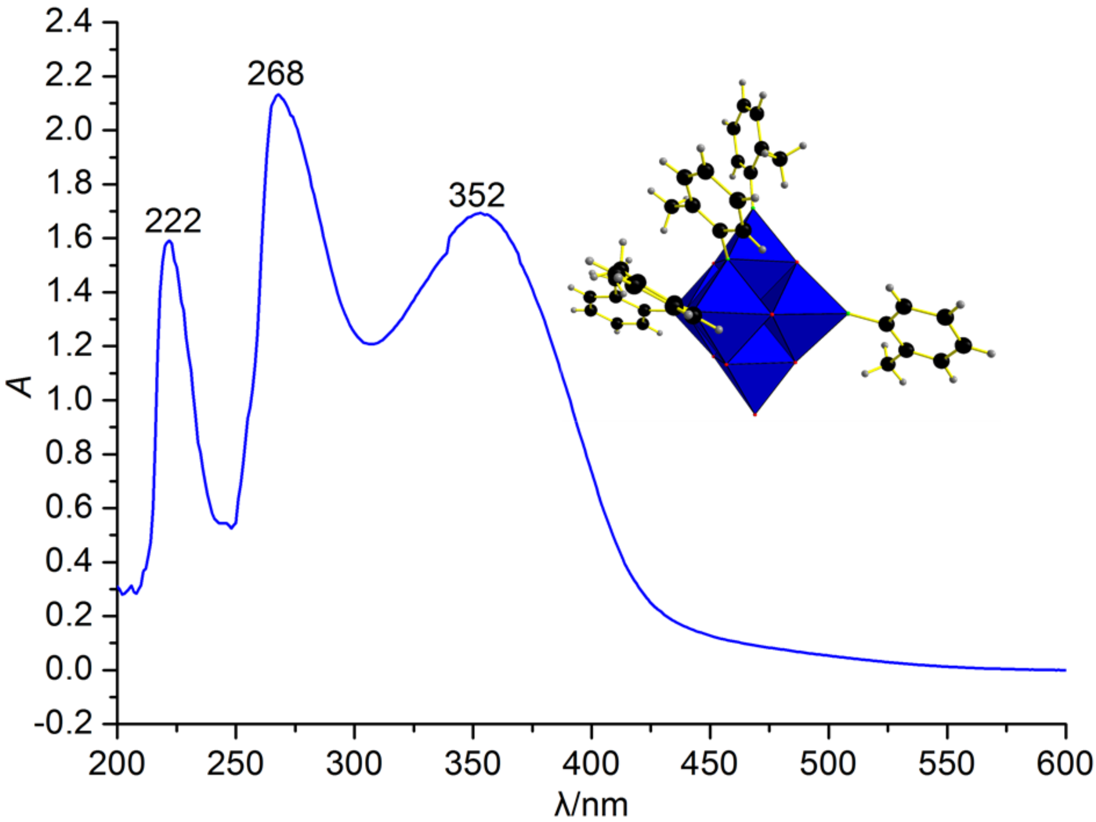

Fig. S2d UV/Vis spectra of compound **4**.

6. ^{1}H NMR data

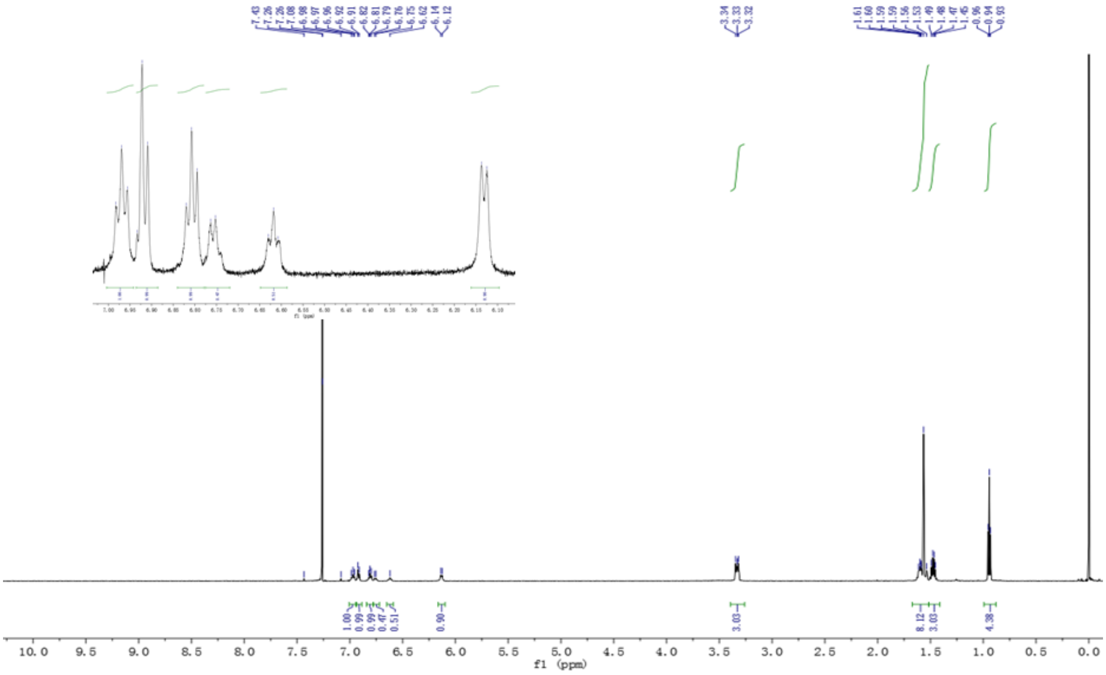

Fig. S3a The ^{1}H NMR spectra of compound **1**.

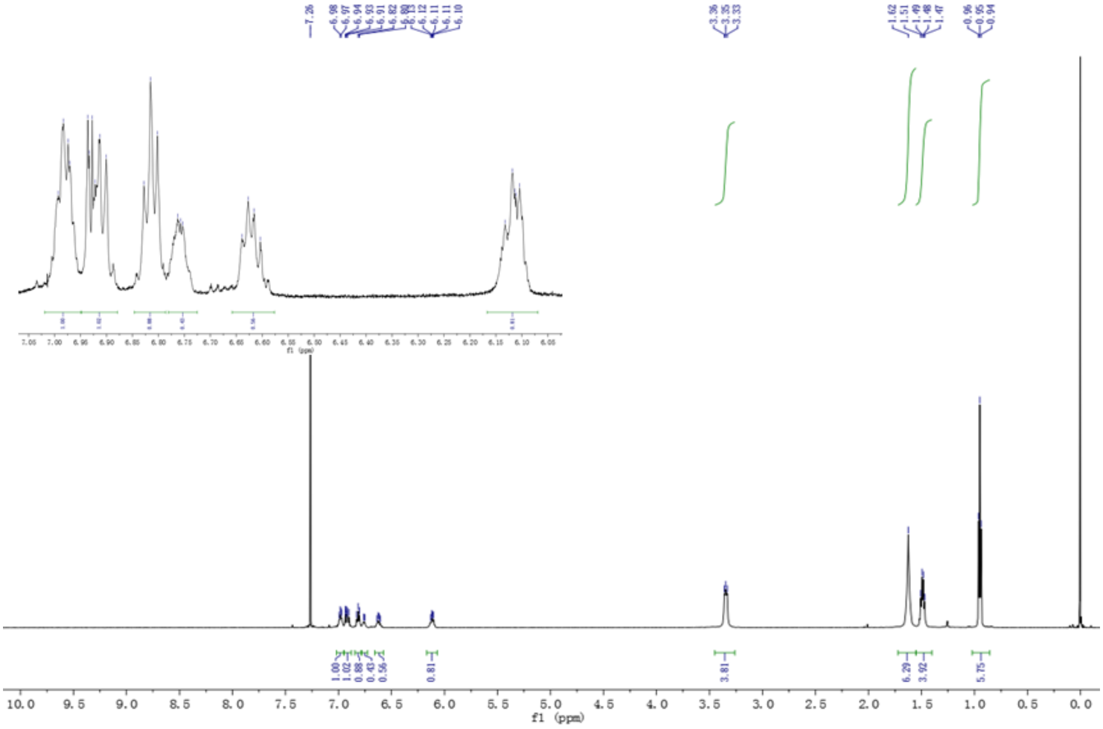

Fig. S3b The ^{1}H NMR spectra of compound **2**.

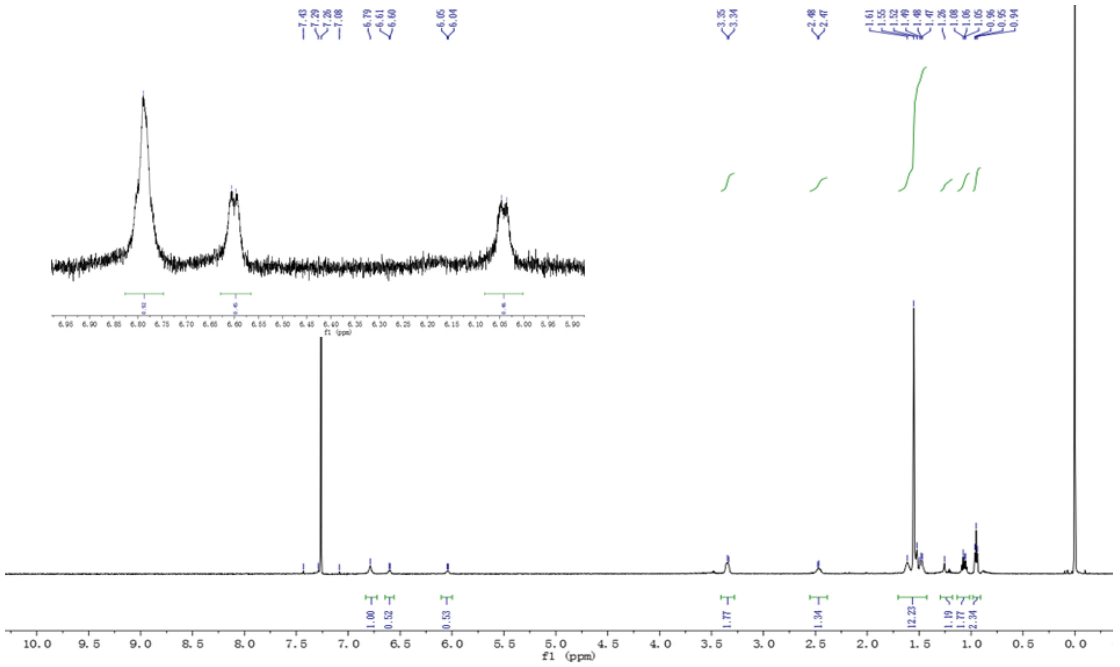

FigS3c. The ^{1}H NMR spectra of compounds **3**.

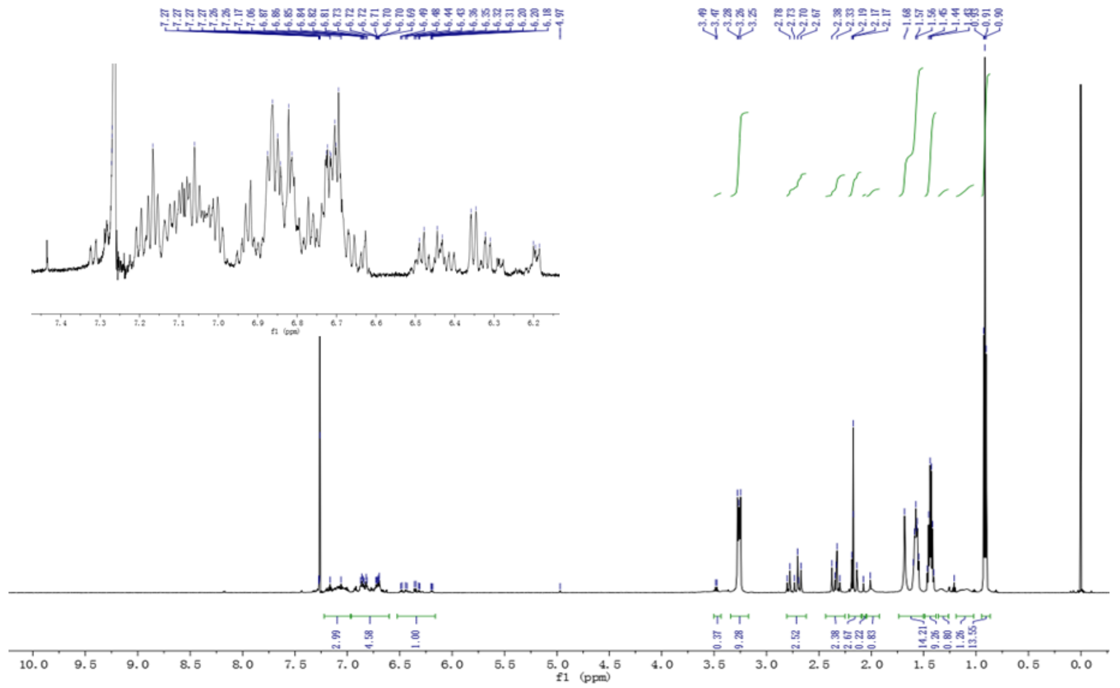

FigS3d. The ^{1}H NMR spectra of compound **4**.

7. ESI mass spectrometry of compounds 1-4

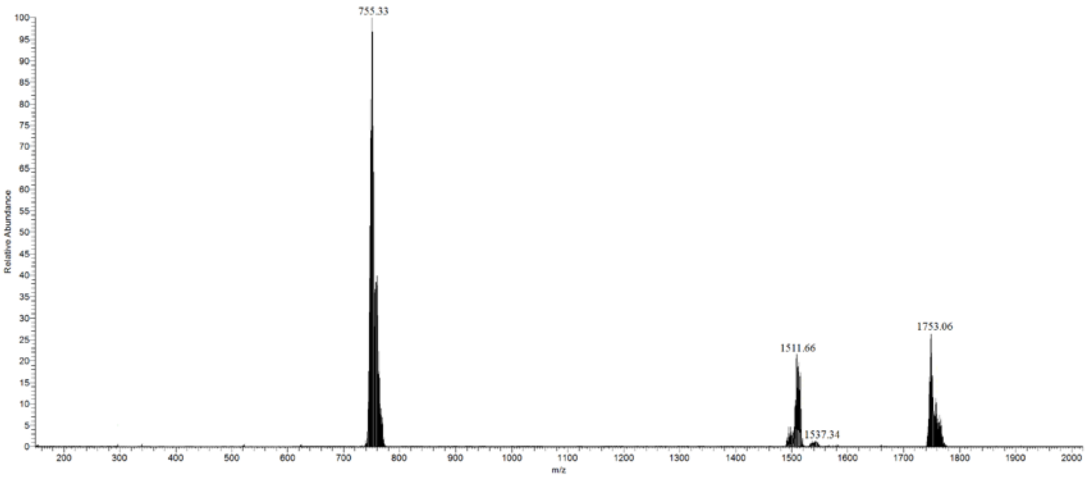

Fig. S4a ESI mass spectrometry of compound **2**.

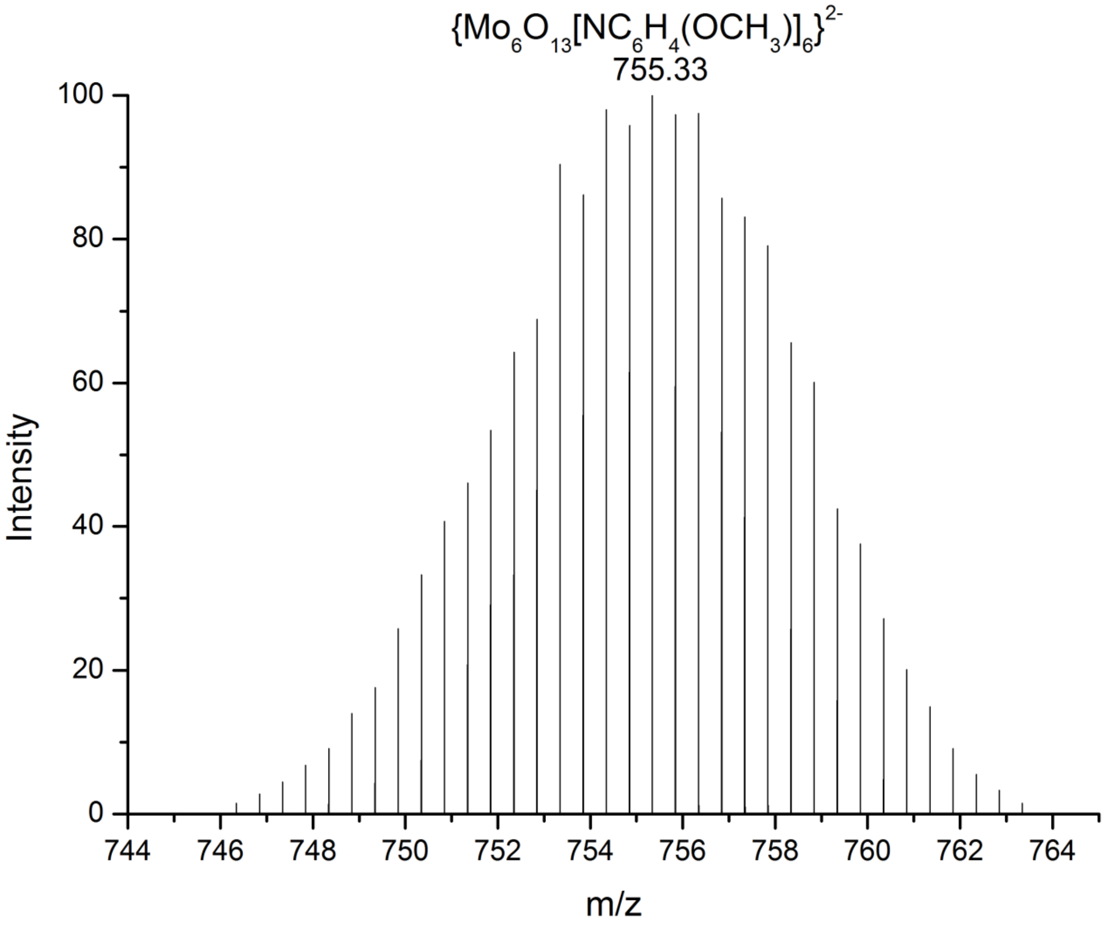

Fig. S4b ESI-MS of compound **2** (100% intensity peak in original size).

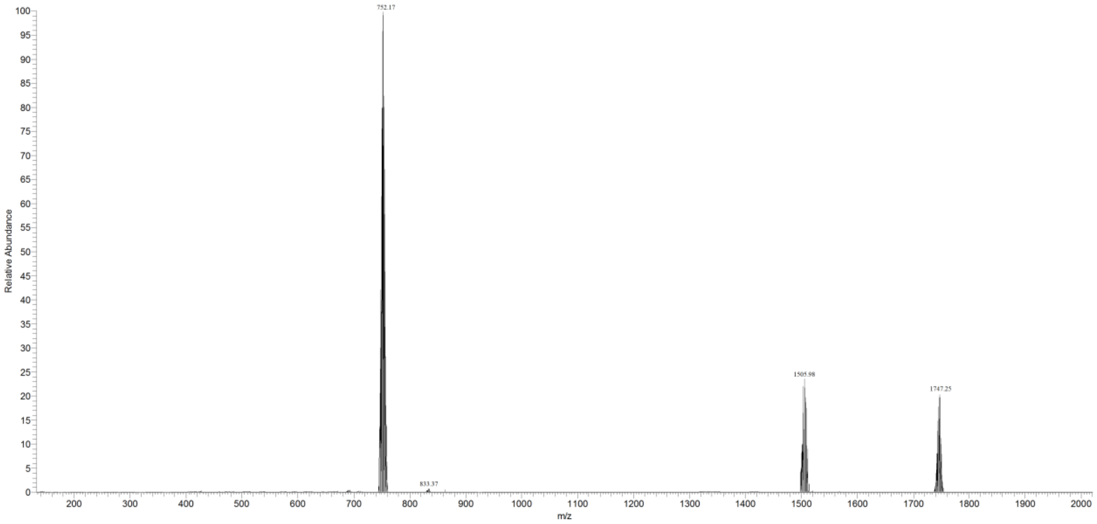

Fig. S4c ESI mass spectrometry of compound **3**.

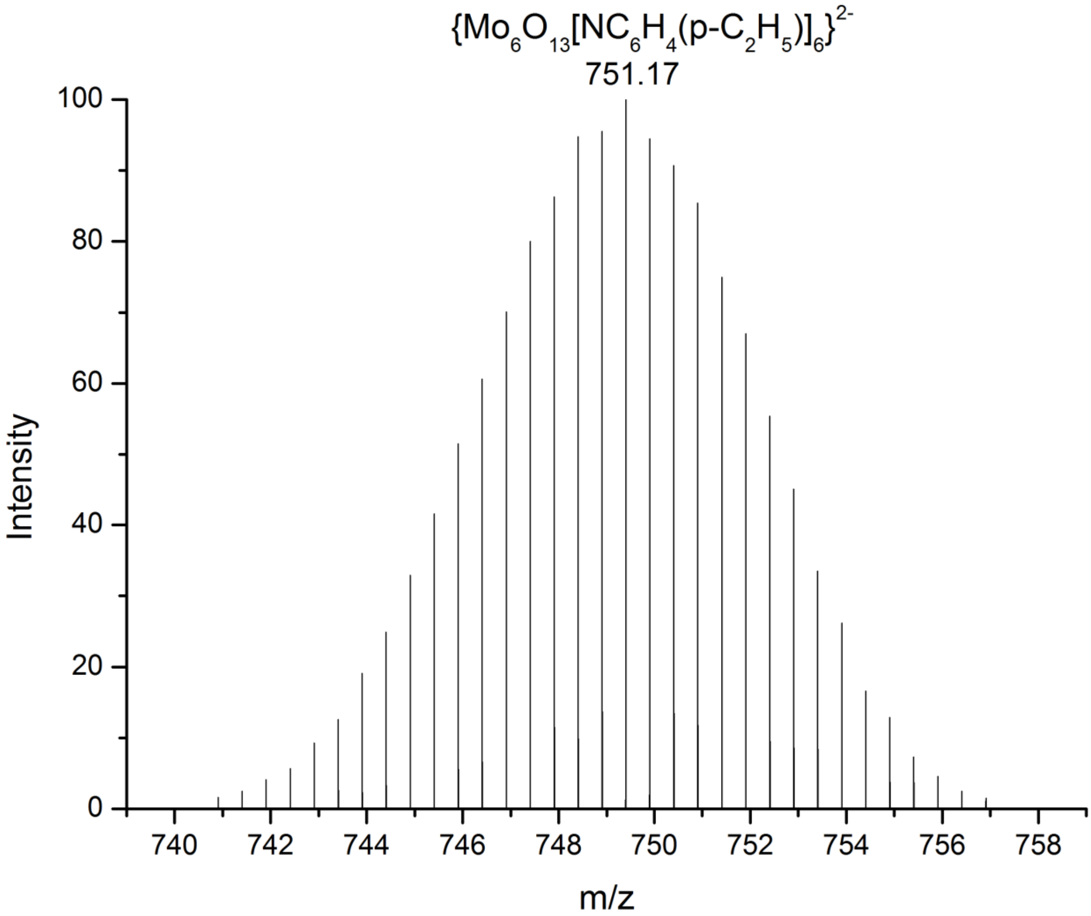

Fig. S4d ESI-MS of compound **3** (100% intensity peak in original size).

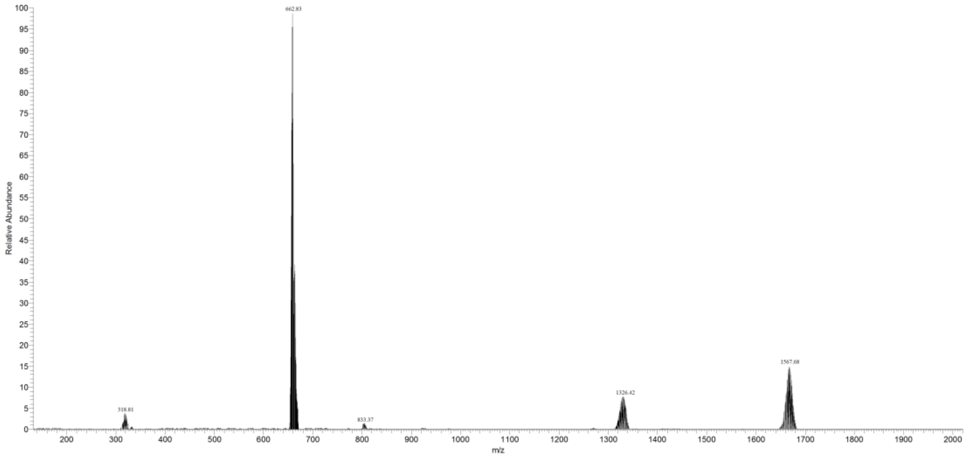

Fig. **S4e** ESI mass spectrometry of compound **4**.

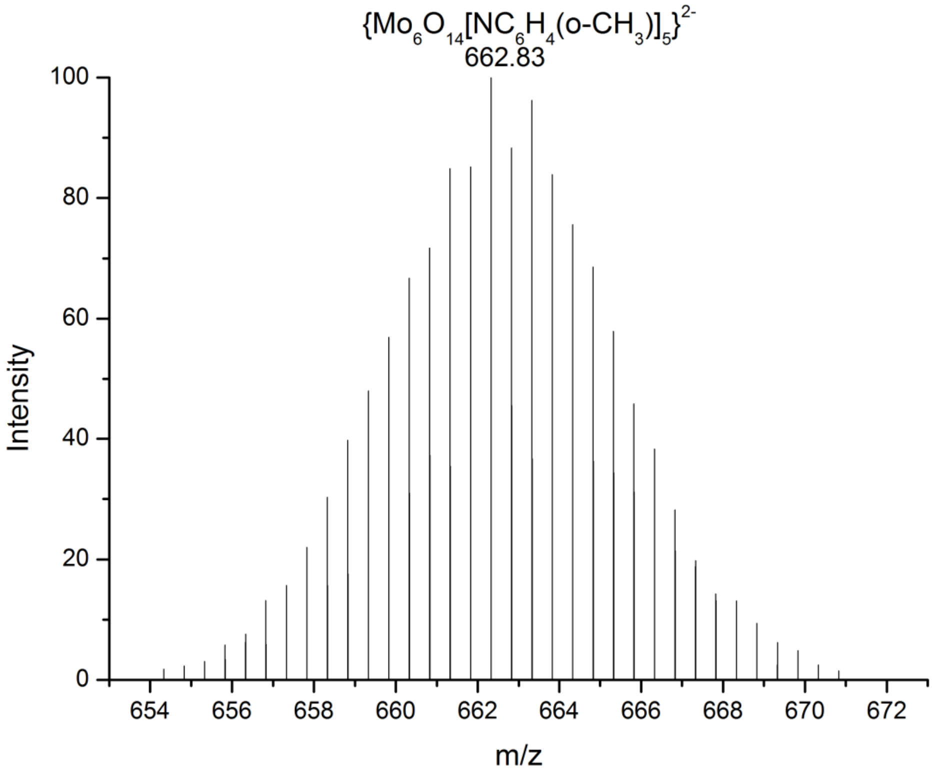

Fig. S4f ESI-MS of compound **3** (100% intensity peak in original size).

8. DFT calculations

Details and results of the DFT calculations

$[Mo_3O_{13}]^{8-}$
b3lyp/gen opt freq pop=full int=grid=ultrafine
O 0
6-31+G(d)
****
Mo 0
LANL2DZ
****
Calculation Type = FREQ
Calculation Method = UB3LYP
Basis Set = Gen
Charge = -8
Spin = Singlet
E(UB3LYP) = -5772.97598225 a.u. a.u.
RMS Gradient Norm = 0.00007893 a.u.
Imaginary Freq = 0
Dipole Moment = 8.4129 Debye
Optimization completed Atomic coordinates

Mo	-1.45629428	-1.22110268	-0.09589316
Mo	-0.31833517	1.82767128	-0.16606904
Mo	1.78864186	-0.65192267	-0.09499247
O	-1.75853767	2.03626340	1.26141107
O	1.73865216	-2.01499760	1.22038014
O	2.62923568	0.58448287	1.25026951
O	0.84342721	2.52986257	1.27030042
O	1.16989415	1.05614127	-0.97320160
O	-0.90464125	-2.53224196	1.20920224
O	-2.67353595	-0.47795965	1.27153810
O	0.01524096	-0.05642297	1.13532086
O	-1.50826871	0.57155467	-0.96242037
O	0.30683663	-1.51414882	-1.08995399
O	3.29181048	-1.17027745	-1.22383833
O	-2.59579083	-2.23557692	-1.31034373
O	-0.62788804	3.46142944	-1.18465231

Mulliken charges with hydrogens summed into heavy atoms:

		1
1	Mo	0.098176
2	Mo	0.083806
3	Mo	0.099381
4	O	-0.734334
5	O	-0.671978
6	O	-0.681767
7	O	-0.700805
8	O	-0.473780
9	O	-0.672996
10	O	-0.675394
11	O	-0.454543
12	O	-0.475914
13	O	-0.491449
14	O	-0.746250
15	O	-0.741947
16	O	-0.760204

Sum of Mulliken charges with hydrogens summed into heavy atoms = -8.00000

$[Mo_3O_{10}(NC_6H_4)_3]^{8-}$
b3lyp/gen opt freq pop=full int=grid=ultrafine
C N O H 0
6-31+G(d)
****
Mo 0
LANL2DZ
****
Calculation Type = FREQ
Calculation Method = UB3LYP
Basis Set = Gen
Charge = -8
Spin = Singlet
E(UB3LYP) = -4539.92934080 a.u.
RMS Gradient Norm = 0.00005431 a.u.
Imaginary Freq = 0
Dipole Moment = 12.0340 Debye
Optimization completed Atomic coordinates

Mo	0.29969000	1.76736800	-1.11878600
Mo	-1.77011200	-0.68116000	-0.55706100
Mo	1.41505600	-1.31347900	-0.77539500
O	-2.76791700	0.26804100	-2.05999000
O	2.47465800	-0.79090300	-2.25689300
O	0.66388700	-2.82197100	-1.87322200
O	-1.90634700	-2.22145300	-1.78785700
O	-0.30626100	-1.48445200	0.26355500
O	1.61323800	1.74606900	-2.53328800
O	-1.04594200	2.27352100	-2.47410500
N	2.55230400	-2.11119800	0.27025100
N	-3.09475500	-1.02099400	0.50158500
O	-0.06845200	-0.23635000	-2.06078900
O	-1.21482000	1.06387400	-0.03317700
N	0.66226900	3.25007500	-0.24032900
C	4.58129700	-3.36408200	0.50164600
H	4.57448800	-3.46994100	-0.42226500
C	3.50582100	-2.55142700	2.49537800
C	3.54641900	-2.67196400	1.10934100
C	2.53771400	5.74222600	1.68633700
H	3.38124900	6.13139600	1.72968400
C	0.98082600	4.25065400	0.65120600
C	1.52363500	6.18454500	2.56241000
H	1.71378100	6.83935800	3.19494600
C	0.00072800	4.70805500	1.53463300
H	-0.86047800	4.36128200	1.48009300
C	2.29408500	4.72347000	0.74966700
C	5.50623400	-3.75149900	2.59216300
H	6.15224600	-4.15760200	3.12381100
C	0.28732100	5.66803800	2.49029000
H	-0.37361200	5.95347300	3.07899100
C	4.51577000	-3.05201600	3.19743900
H	4.54379700	-2.92065000	4.11764600
C	-4.46223800	-1.07138200	2.48623200
H	-3.71497600	-0.80007700	2.96883200
C	-5.70638800	-1.26136300	3.14737800
H	-5.78820500	-1.12858700	4.06414200

C	-4.36364000	-1.29342500	1.10706900
C	-6.78956100	-1.64965700	2.38321700
H	-7.61744600	-1.79472800	2.78132700
C	-6.62999000	-1.82067200	1.01705900
H	-7.38421900	-2.02832800	0.51429200
C	-5.48386500	-1.70886700	0.38595100
C	5.59945700	-3.88887200	1.21653900
H	6.31895900	-4.31607200	0.81057500
O	1.49918600	0.52057400	-0.02820000
H	-5.38579100	-1.90016600	-0.52913700
H	2.98430700	4.41366500	0.22620400
H	2.75096900	-2.14896200	2.96047300

Mulliken charges with hydrogens summed into heavy atoms:

1	Mo	0.449803
2	Mo	0.480517
3	Mo	0.450963
4	O	-0.402304
5	O	-0.419452
6	O	-0.431352
7	O	-0.401886
8	O	-0.378572
9	O	-0.441213
10	O	-0.435617
11	N	-0.437924
12	N	-0.437910
13	O	-0.334566
14	O	-0.379443
15	N	-0.450383
16	C	-0.220670
18	C	-0.211900
19	C	0.002746
20	C	-0.311693
22	C	0.016304
23	C	-0.320665
25	C	-0.223978
27	C	-0.245455
28	C	-0.326624
30	C	-0.273428
32	C	-0.255135
34	C	-0.221524
36	C	-0.311196
38	C	0.009915
39	C	-0.333785
41	C	-0.271303
43	C	-0.235452
44	C	-0.320179
46	O	-0.376637

Sum of Mulliken charges with hydrogens summed into heavy atoms = -8.00000

$[Mo_3O_7(NC_6H_5)_3(\mu_2\text{-}NC_6H_5)_3]^{8-}$
b3lyp/gen opt freq pop=full int=grid=ultrafine
C N O H 0
6-31+G(d)
****
Mo 0
LANL2DZ
****

Calculation Type = FREQ
Calculation Method = UB3LYP
Basis Set = Gen
Charge = -8
Spin = Singlet
E(UB3LYP) = -4644.47931261 a.u.
RMS Gradient Norm = 0.00008632 a.u.
Imaginary Freq = 0
Dipole Moment = 11.7746 Debye
Optimization completed Atomic coordinates

Mo	0.15479400	1.81195200	-1.32464100
Mo	-1.68460600	-0.80876200	-1.27266300
Mo	1.51249600	-1.09289300	-1.30443400
O	-0.02183500	-0.03231200	-2.28055200
O	1.03574700	-2.61842900	-2.61251300
N	-1.33134300	0.99587900	-0.33294800
N	0.40279800	3.27410700	-0.37150100
O	-1.74140700	-2.26853800	-2.66711700
N	1.55345600	0.62560300	-0.36135200
O	1.68294800	2.15410900	-2.71112700
O	2.74674400	-0.40803400	-2.74609600
N	2.67589800	-2.01150600	-0.36433200
O	-1.07783200	2.55083600	-2.75181800
O	-2.76739700	0.33617100	-2.63688800
N	-0.22040800	-1.67319700	-0.31104900
N	-3.07310700	-1.29748400	-0.30746900
C	-5.14637800	-0.54345800	0.65294200
H	-4.88763000	0.34400400	0.53846900
C	-4.25671500	-1.58722000	0.32273500
C	-2.31879700	0.83351900	1.89519100
H	-1.88919500	0.02115200	2.02789900
C	-3.14994600	1.34218600	2.87749400
H	-3.28453800	0.86855100	3.66622200
C	2.41862000	1.04945300	0.64856300
C	4.65056300	1.56364300	1.44176600
H	5.56599400	1.61271600	1.29134700
C	1.90469500	1.49996700	1.87324300
H	0.98519200	1.50739600	2.02012600
C	-4.64900300	-2.90943100	0.53124400
H	-4.06677600	-3.60505900	0.32751500
C	2.37921700	5.90305200	1.27125600
H	3.26043500	6.06823700	1.52410000
C	-2.11950600	1.55809500	0.67745300
C	2.04276500	4.68304300	0.69673400
H	2.68991800	4.02333600	0.59047900
C	-0.28364600	-2.57583800	0.74329300
C	0.74010500	4.45439900	0.28299900
C	2.77463500	1.94005300	2.87204300
H	2.43546800	2.20996500	3.69351400
C	3.59454800	-2.81731900	0.30374300
C	4.91570700	-2.43355300	0.48276600
H	5.18474300	-1.58075800	0.22702000
C	0.41937000	-2.39011700	1.94726600
H	0.95309900	-1.63555800	2.04664700
C	-0.40988300	-4.44534900	2.83037800
H	-0.45467200	-5.06745800	3.51922300
C	0.34154000	-3.28350400	2.97213000

H	0.79286100	-3.11540700	3.76870400
C	-1.06029100	-3.73780800	0.62381900
H	-1.55586200	-3.88577200	-0.15058500
C	-6.78214600	-2.13674500	1.32373200
H	-7.63796000	-2.32155000	1.63547800
C	-0.20641600	5.42611600	0.51842500
H	-1.09085300	5.25519600	0.28571700
C	0.10674100	6.64206100	1.08382100
H	-0.54491900	7.29519800	1.20448600
C	3.79728600	1.07565300	0.44812500
H	4.15208400	0.76557500	-0.35185000
C	4.12133900	1.97466000	2.64959100
H	4.68777800	2.27919700	3.31856700
C	-5.92036800	-3.19173700	1.04639600
H	-6.18418500	-4.06995000	1.19907500
C	-1.09434600	-4.66060800	1.63981800
H	-1.58424900	-5.44386400	1.52961000
C	-2.75260700	2.74096100	0.51724200
H	-2.63106800	3.22271400	-0.26819300
C	5.83277900	-3.29389500	1.03552600
H	6.71653000	-3.02594100	1.14811800
C	-6.40367100	-0.83940600	1.15048400
H	-6.99088300	-0.15140100	1.36676000
C	3.19887300	-4.09911500	0.70495200
H	2.32017300	-4.37921500	0.57936600
C	5.44149300	-4.54222700	1.42019100
H	6.06738900	-5.13307200	1.77524600
C	1.41981100	6.86852800	1.46960300
H	1.65204600	7.67849600	1.86576300
C	-3.77978300	2.56069700	2.67803300
H	-4.33461200	2.90057800	3.34347700
C	4.11578000	-4.94028700	1.28519400
H	3.85023600	-5.77824800	1.58844200
C	-3.60306900	3.26526400	1.53735800
H	-4.03183500	4.08183100	1.41840900

Mulliken charges with hydrogens summed into heavy atoms:

1	Mo	-11.558975
2	Mo	-11.178759
3	Mo	-10.557201
4	O	7.855999
5	O	3.190679
6	N	9.772226
7	N	5.595481
8	O	2.228053
9	N	9.998500
10	O	3.509156
11	O	1.843636
12	N	5.487971
13	O	2.194198
14	O	3.453725
15	N	7.604150
16	N	5.682260
17	C	-1.911982
19	C	3.473862
20	C	4.052016
22	C	0.898270
24	C	-25.402617

25	C	2.442784
27	C	2.951699
29	C	0.075735
31	C	-0.002577
33	C	-26.509800
34	C	-0.378229
36	C	-23.086563
37	C	3.268889
38	C	0.182574
40	C	3.067152
41	C	1.226277
43	C	1.910835
45	C	-0.850410
47	C	0.068098
49	C	5.664175
51	C	0.573694
53	C	0.485276
55	C	-2.618267
57	C	4.781158
59	C	-0.968891
61	C	-2.264749
63	C	2.471071
65	C	4.048439
67	C	-2.736477
69	C	1.019800
71	C	-1.451065
73	C	0.546466
75	C	0.272345
77	C	-0.660382
79	C	0.075925
81	C	2.164370

Sum of Mulliken charges with hydrogens summed into heavy atoms = -8.00000

Compound**1** $[Mo_6O_{13}(NAr)_3(\mu_2\text{-}NAr)_3]^{2-}$
b3lyp/gen opt freq pop=full NMR int=grid=ultrafine
C N O H 0
6-31+G(d)
****
Mo 0
LANL2DZ
****
Calculation Type = FREQ
Calculation Method = UB3LYP
Basis Set = Gen
Charge = -2
Spin = Singlet
E(UB3LYP) = -7524.98622905 a.u.
RMS Gradient Norm = 0.00008543 a.u.
Imaginary Freq = 0
Dipole Moment = 6.1645 Debye
Optimization completed Atomic coordinates

Mo	1.32880600	-1.13729800	0.81771200
Mo	-0.02737500	0.08249500	-1.88092200
Mo	-1.56077400	0.36463500	0.98363000
Mo	0.21423600	-3.20521700	-1.47378000
Mo	-2.78983200	-1.65483600	-1.36654400
Mo	-1.41365300	-2.89210500	1.46857400

O	-0.59947800	-3.99277000	0.17469400
O	-3.08232400	-0.16702700	-0.28963700
N	1.68573800	0.00986200	-0.72981200
O	-1.75685100	-0.55828100	-2.56175100
O	-1.57592400	-3.08652300	-2.05858400
N	0.56159300	1.17596800	-3.11614500
O	1.61625500	-2.75072300	-0.25270100
N	0.23882500	0.38653400	1.72082400
O	-0.58784200	-1.16724100	-0.20795600
O	0.66273300	-1.76241700	-2.51584300
O	-2.90119300	-2.77326200	0.14978200
O	0.81655300	-4.57640000	-2.28510500
O	-1.99830900	-3.95319400	2.65231300
N	2.81291500	-0.92670400	1.72439800
O	0.26496100	-2.34710200	2.04880800
O	-2.10717300	-1.16743200	2.02022500
O	-4.24539300	-1.90944600	-2.19909000
C	-1.22755400	2.70736900	-1.00242700
C	2.94796900	0.48683500	-1.13197500
N	-0.88169700	1.36529600	-0.68346800
C	0.79076200	1.18904200	2.71808300
C	3.81673400	-0.59022400	2.59713000
C	3.19980400	1.85885900	-1.21249200
H	2.53056700	2.46350700	-0.99136600
C	-2.24262100	2.97503600	-1.90964700
H	-2.67815400	2.27404800	-2.33689900
C	5.45371800	1.42430800	-1.92597800
H	6.30234700	1.72886500	-2.15777200
C	3.62263400	-0.67633400	3.97146200
H	2.81291900	-0.99185200	4.29971200
C	1.92269300	1.98186900	2.49373800
H	2.29660200	2.02298200	1.64441900
N	-2.30853000	1.70718800	1.81260200
C	5.05683100	-0.14287100	2.09959000
H	5.21598300	-0.10803000	1.18405200
C	3.95946900	-0.38292200	-1.50474600
H	3.80236200	-1.29833600	-1.50004000
C	-0.53918700	3.75859800	-0.42413000
H	0.14864600	3.60540800	0.18025800
C	-1.91476000	5.33086300	-1.61815500
H	-2.14682400	6.21130500	-1.81708600
C	2.48912400	2.71234500	3.53941700
H	3.25365000	3.21998600	3.38389000
C	-3.66093200	3.63330800	1.50618500
H	-3.63104300	3.49860700	0.58641700
C	5.20084500	0.09060100	-1.88875300
H	5.87114600	-0.50942300	-2.12365300
C	-0.91464900	5.06935700	-0.78056000
H	-0.42662800	5.77602900	-0.42561600
C	6.03749200	0.24421400	3.00978100
H	6.85462500	0.55990200	2.69807900
C	1.17614000	2.09073400	-3.96748500
C	-2.61244500	4.27786600	-2.18487500
H	-3.32990100	4.44787600	-2.75391100
C	0.55767800	3.30221500	-4.23366000
H	-0.28310700	3.48796500	-3.87803900
C	-2.98783100	2.79418200	2.33297000

C	0.23997800	1.17101200	4.01140400
H	-0.51376700	0.65196000	4.18301300
C	4.61430500	-0.30218700	4.84460600
H	4.47391200	-0.36905000	5.76329400
C	5.80835600	0.16638700	4.36616200
H	6.47178200	0.43682400	4.96088600
C	1.20521600	4.22046900	-5.04540200
H	0.82902700	5.06667700	-5.17768500
C	4.46234500	2.32825700	-1.62526200
H	4.62373800	3.24218200	-1.69256700
C	1.94349000	2.68919700	4.77534800
H	2.32074600	3.18948800	5.46391500
C	0.80053300	1.91340700	5.02728100
H	0.42431100	1.89884800	5.87800500
C	-4.50709200	5.13458300	3.18456400
H	-4.91792500	5.93680300	3.41949800
C	-4.39173100	4.68157500	2.02696100
H	-4.89688800	5.13619300	1.39465600
C	2.41087900	1.79555800	-4.51984300
H	2.84915300	1.01253200	-4.27067600
C	-3.83689100	4.11643600	4.16733400
H	-4.14018600	3.91724100	5.02252700
C	-2.80515700	3.61123000	3.60056000
H	-1.95770300	3.73866800	3.96130900
C	2.40336100	3.88604800	-5.63703900
H	2.81327100	4.48119400	-6.22349700
C	2.99849000	2.64018000	-5.39164600
H	3.79015400	2.39243700	-5.83026600

Mulliken charges with hydrogens summed into heavy atoms:

1	Mo	-13.535571
2	Mo	-17.448901
3	Mo	-18.920689
4	Mo	18.130434
5	Mo	18.234115
6	Mo	18.927782
7	O	-2.770058
8	O	0.056600
9	N	13.658153
10	O	-0.806703
11	O	-2.702914
12	N	7.671590
13	O	-1.048323
14	N	15.449651
15	O	-7.541916
16	O	0.510603
17	O	-2.843181
18	O	-8.018121
19	O	-8.868039
20	N	6.706908
21	O	-0.058001
22	O	-0.121904
23	O	-8.737505
24	C	-16.023076
25	C	-19.844816
26	N	14.687176
27	C	-23.323808
28	C	-1.796785

29	C	2.322161
31	C	4.034526
33	C	-0.306631
35	C	2.950025
37	C	0.801481
39	N	7.430300
40	C	0.214951
42	C	3.795794
44	C	0.661576
46	C	-0.246468
48	C	0.451385
50	C	-0.473987
52	C	0.517906
54	C	-0.005547
56	C	-0.835807
58	C	2.393429
59	C	0.576242
61	C	0.071101
63	C	3.349325
64	C	7.919842
66	C	-0.025316
68	C	0.397176
70	C	-1.030149
72	C	0.710107
74	C	-0.240346
76	C	1.141993
78	C	0.233236
80	C	-1.507982
82	C	1.715586
84	C	-0.090108
86	C	1.527314
88	C	0.375056
90	C	-0.450872

Sum of Mulliken charges with hydrogens summed into heavy atoms = -2.00000

$\{Mo_3O_{10}[NC_6H_4(o\text{-}CH_3)]_3\}^{8-}$
b3lyp/gen opt freq pop=full int=grid=ultrafine
C N O H 0
6-31+G(d)
****
Mo 0
LANL2DZ
****
Calculation Type = FREQ
Calculation Method = UB3LYP
Basis Set = Gen
Charge = -8
Spin = Singlet
E(UB3LYP) = -4416.38056050 a.u.
RMS Gradient Norm = 0.00007741 a.u.
Imaginary Freq = 0
Dipole Moment = 10.4724 Debye
Optimization completed Atomic coordinates

Mo	0.15479400	1.81195200	-1.32464100
Mo	-1.68460600	-0.80876200	-1.27266300
Mo	1.51249600	-1.09289300	-1.30443400
O	-0.02183500	-0.03231200	-2.28055200

O	1.03574700	-2.61842900	-2.61251300
N	-1.33134300	0.99587900	-0.33294800
N	0.40279800	3.27410700	-0.37150100
O	-1.74140700	-2.26853800	-2.66711700
N	1.55345600	0.62560300	-0.36135200
O	1.68294800	2.15410900	-2.71112700
O	2.74674400	-0.40803400	-2.74609600
N	2.67589800	-2.01150600	-0.36433200
O	-1.07783200	2.55083600	-2.75181800
O	-2.76739700	0.33617100	-2.63688800
N	-0.22040800	-1.67319700	-0.31104900
N	-3.07310700	-1.29748400	-0.30746900
C	-5.14637800	-0.54345800	0.65294200
H	-4.88763000	0.34400400	0.53846900
C	-4.25671500	-1.58722000	0.32273500
C	-2.31879700	0.83351900	1.89519100
H	-1.88919500	0.02115200	2.02789900
C	-3.14994600	1.34218600	2.87749400
H	-3.28453800	0.86855100	3.66622200
C	2.41862000	1.04945300	0.64856300
C	4.65056300	1.56364300	1.44176600
H	5.56599400	1.61271600	1.29134700
C	1.90469500	1.49996700	1.87324300
H	0.98519200	1.50739600	2.02012600
C	-4.64900300	-2.90943100	0.53124400
H	-4.06677600	-3.60505900	0.32751500
C	2.37921700	5.90305200	1.27125600
H	3.26043500	6.06823700	1.52410000
C	-2.11950600	1.55809500	0.67745300
C	2.04276500	4.68304300	0.69673400
H	2.68991800	4.02333600	0.59047900
C	-0.28364600	-2.57583800	0.74329300
C	0.74010500	4.45439900	0.28299900
C	2.77463500	1.94005300	2.87204300
H	2.43546800	2.20996500	3.69351400
C	3.59454800	-2.81731900	0.30374300
C	4.91570700	-2.43355300	0.48276600
H	5.18474300	-1.58075800	0.22702000
C	0.41937000	-2.39011700	1.94726600
H	0.95309900	-1.63555800	2.04664700
C	-0.40988300	-4.44534900	2.83037800
H	-0.45467200	-5.06745800	3.51922300
C	0.34154000	-3.28350400	2.97213000
H	0.79286100	-3.11540700	3.76870400
C	-1.06029100	-3.73780800	0.62381900
H	-1.55586200	-3.88577200	-0.15058500
C	-6.78214600	-2.13674500	1.32373200
H	-7.63796000	-2.32155000	1.63547800
C	-0.20641600	5.42611600	0.51842500
H	-1.09085300	5.25519600	0.28571700
C	0.10674100	6.64206100	1.08382100
H	-0.54491900	7.29519800	1.20448600
C	3.79728600	1.07565300	0.44812500
H	4.15208400	0.76557500	-0.35185000
C	4.12133900	1.97466000	2.64959100
H	4.68777800	2.27919700	3.31856700
C	-5.92036800	-3.19173700	1.04639600

H	-6.18418500	-4.06995000	1.19907500
C	-1.09434600	-4.66060800	1.63981800
H	-1.58424900	-5.44386400	1.52961000
C	-2.75260700	2.74096100	0.51724200
H	-2.63106800	3.22271400	-0.26819300
C	5.83277900	-3.29389500	1.03552600
H	6.71653000	-3.02594100	1.14811800
C	-6.40367100	-0.83940600	1.15048400
H	-6.99088300	-0.15140100	1.36676000
C	3.19887300	-4.09911500	0.70495200
H	2.32017300	-4.37921500	0.57936600
C	5.44149300	-4.54222700	1.42019100
H	6.06738900	-5.13307200	1.77524600
C	1.41981100	6.86852800	1.46960300
H	1.65204600	7.67849600	1.86576300
C	-3.77978300	2.56069700	2.67803300
H	-4.33461200	2.90057800	3.34347700
C	4.11578000	-4.94028700	1.28519400
H	3.85023600	-5.77824800	1.58844200
C	-3.60306900	3.26526400	1.53735800
H	-4.03183500	4.08183100	1.41840900

Mulliken charges with hydrogens summed into heavy atoms:

1	Mo	-11.558975
2	Mo	-11.178759
3	Mo	-10.557201
4	O	7.855999
5	O	3.190679
6	N	9.772226
7	N	5.595481
8	O	2.228053
9	N	9.998500
10	O	3.509156
11	O	1.843636
12	N	5.487971
13	O	2.194198
14	O	3.453725
15	N	7.604150
16	N	5.682260
17	C	-1.911982
19	C	3.473862
20	C	4.052016
22	C	0.898270
24	C	-25.402617
25	C	2.442784
27	C	2.951699
29	C	0.075735
31	C	-0.002577
33	C	-26.509800
34	C	-0.378229
36	C	-23.086563
37	C	3.268889
38	C	0.182574
40	C	3.067152
41	C	1.226277
43	C	1.910835
45	C	-0.850410
47	C	0.068098

49	C	5.664175
51	C	0.573694
53	C	0.485276
55	C	-2.618267
57	C	4.781158
59	C	-0.968891
61	C	-2.264749
63	C	2.471071
65	C	4.048439
67	C	-2.736477
69	C	1.019800
71	C	-1.451065
73	C	0.546466
75	C	0.272345
77	C	-0.660382
79	C	0.075925
81	C	2.164370

Sum of Mulliken charges with hydrogens summed into heavy atoms = -8.00000

$\{Mo_3O_7[NC_6H_4(o\text{-}CH_3)]_3[\mu_2\text{-}NC_6H_4(o\text{-}CH_3)]_3\}^{8-}$
b3lyp/gen opt freq pop=full int=grid=ultrafine
C N O H 0
6-31+G(d)
****
Mo 0
LANL2DZ
****
Calculation Type = FREQ
Calculation Method = UB3LYP
Basis Set = Gen
Charge = -8
Spin = Singlet
E(UB3LYP) = -4125.70147170 a.u.
RMS Gradient Norm = 0.00006742 a.u.
Imaginary Freq = 0
Dipole Moment = 5.8278 Debye
Optimization completed Atomic coordinates

Mo	0.44613200	1.67762400	-1.69375100
Mo	-1.89640700	-0.48798800	-1.04758300
Mo	1.15756700	-1.53126000	-1.46902500
O	-2.84373300	0.62124500	-2.47151100
O	2.18988900	-1.11787800	-3.00333400
O	0.14911500	-2.89983300	-2.54376200
O	-2.30844700	-1.96631900	-2.29284700
O	1.66085400	1.51681500	-3.18548600
O	-0.89599100	2.39034900	-2.95650000
N	2.23660500	-2.49776800	-0.50746000
N	-3.19100800	-0.67491400	0.08377300
O	-0.24056700	-0.23619200	-2.64429600
N	1.52931400	0.25679500	-0.69844800
N	1.05398500	3.07745700	-0.81480100
C	4.08983200	-4.01367800	-0.42006700
H	4.01540800	-4.09524500	-1.34346900
C	3.25001100	-3.11432800	1.64824000
C	3.19375000	-3.20550300	0.26050900
C	3.35425700	5.25164700	1.03500800
H	4.24333400	5.52445500	1.03345900

C	1.55458100	4.00507100	0.07199500
C	2.46090400	5.80303000	1.97809000
H	2.77322200	6.41132900	2.60854000
C	0.69728900	4.56671700	1.02060100
H	-0.20425000	4.33853300	1.01274400
C	3.91444400	3.67123100	-0.82563400
H	4.44116600	4.35730300	-1.24229000
H	3.45100300	3.16954600	-1.50036000
H	4.48895300	3.08438300	-0.32855800
C	2.92274400	4.29718900	0.09832500
C	5.07422500	-4.57085500	1.60388600
H	5.68984800	-5.07175300	2.08866400
C	1.16454900	5.45678400	1.97266900
H	0.58294500	5.81289900	2.60496200
C	4.22292800	-3.76120700	2.27947400
H	4.32153900	-3.65708800	3.19830900
C	-4.43563800	-0.59183200	2.14662600
H	-3.63211500	-0.43372100	2.58737600
C	-5.65360100	-0.63128600	2.87883200
H	-5.66367300	-0.51113300	3.80091800
C	2.28843600	-2.41047800	2.39398500
H	2.65226700	-1.56566600	2.66903100
H	2.04698200	-2.92252600	3.16926900
H	1.50932000	-2.26243500	1.85294100
C	-4.44769200	-0.79143900	0.76057900
C	4.40804100	0.15820100	-0.50805100
H	4.34451900	-0.78742900	-0.66104600
H	5.32385600	0.39508600	-0.34467500
H	4.08855200	0.62957000	-1.28097700
C	-5.62326700	-1.33097400	-1.40063100
H	-5.11120600	-0.65384600	-1.84894400
H	-6.51976000	-1.33408800	-1.74400600
H	-5.22002500	-2.18915700	-1.55083700
C	-6.82140500	-0.85398100	2.17554100
H	-7.63678300	-0.89770300	2.62071400
C	-6.76556100	-1.01142000	0.79960800
H	-7.56863500	-1.10505000	0.34019300
C	-5.65328900	-1.03717000	0.10205100
C	1.42848200	0.95426400	1.72498500
H	0.50527400	1.03850000	1.65016400
C	2.22142000	0.56769600	0.58118600
C	4.15910500	0.90038500	1.88649000
H	5.08395800	0.99439200	1.91253600
C	2.04226600	1.19198500	2.90487100
H	1.52982400	1.39299900	3.65441700
C	5.06838000	-4.68593800	0.22308100
H	5.69956100	-5.19472100	-0.23270500
C	3.59219000	0.52163100	0.65942700
C	3.49253900	1.13881000	3.02261400
H	3.92784600	1.26262400	3.83504400
N	-1.08337000	1.15485400	-0.52991000
C	-1.50649600	1.81634200	0.46315200
C	-1.09421500	1.47953000	1.75236500
C	-2.38499300	2.88199700	0.26790200
C	-1.56058300	2.20838000	2.84640900
C	-2.85128500	3.61090900	1.36196300
C	-2.43908000	3.27403500	2.65115900

H	-1.23550700	1.94281300	3.86308800
H	-3.54401400	4.45141400	1.20802100
H	-2.80688600	3.84885000	3.51386100
N	-0.50803800	-1.49782900	-0.33050700
C	-0.67398200	-2.08227700	0.78019400
C	-0.51875100	-1.37344200	1.97138900
C	-1.01207600	-3.43510500	0.81258400
C	-0.70151700	-2.01755000	3.19506200
C	-1.19493600	-4.07918600	2.03628000
C	-1.03961200	-3.37037800	3.22745200
H	-0.57910600	-1.45862600	4.13445400
H	-1.46168800	-5.14605400	2.06187300
H	-1.18376600	-3.87837800	4.19238900
H	-0.26918900	-0.37442000	1.94744000
H	-0.42650700	0.66973900	1.90070800
C	-2.84014500	3.25389500	-1.15551900
H	-2.02241900	3.69253000	-1.68827000
H	-3.16743600	2.37285200	-1.66693300
H	-3.64682300	3.95470000	-1.10035500
C	-1.18351500	-4.21770600	-0.50261300
H	-1.81906700	-3.66762600	-1.16472000
H	-0.22706200	-4.35839900	-0.96120100
H	-1.62353300	-5.17084900	-0.29572500

Mulliken charges with hydrogens summed into heavy atoms:

1	Mo	0.597522
2	Mo	0.692799
3	Mo	0.616453
4	O	-0.387784
5	O	-0.399267
6	O	-0.390428
7	O	-0.378820
8	O	-0.413575
9	O	-0.392804
10	N	-0.416629
11	N	-0.399803
12	O	-0.330500
13	N	-0.424266
14	N	-0.434496
15	C	-0.148805
17	C	-0.018567
18	C	0.003154
19	C	-0.233844
21	C	0.015933
22	C	-0.281833
24	C	-0.101718
26	C	-0.097881
30	C	-0.027529
31	C	-0.297756
33	C	-0.179241
35	C	-0.189862
37	C	-0.100788
39	C	-0.219449
41	C	-0.036177
45	C	-0.005477
46	C	-0.071776
50	C	-0.096311
54	C	-0.317057

56	C	-0.229538
58	C	-0.053991
59	C	-0.000799
61	C	0.064389
62	C	-0.154308
64	C	-0.123064
66	C	-0.236859
68	C	-0.024785
69	C	-0.224917
71	N	-0.463246
72	C	0.088366
73	C	-0.017738
74	C	-0.021446
75	C	-0.135034
76	C	-0.186369
77	C	-0.236765
81	N	-0.456566
82	C	0.081742
83	C	-0.024797
84	C	-0.023968
85	C	-0.132430
86	C	-0.185723
87	C	-0.245246
93	C	-0.102793
97	C	-0.087533

Sum of Mulliken charges with hydrogens summed into heavy atoms = -8.00000

$\{Mo_3O_9[NC_6H_4(o\text{-}CH_3)]_4[\mu_2\text{-}NC_6H_4(o\text{-}CH_3)]\}^{8-}$
b3lyp/gen opt freq pop=full int=grid=ultrafine
C N O H 0
6-31+G(d)
****
Mo 0
LANL2DZ
****
Calculation Type = FREQ
Calculation Method = UB3LYP
Basis Set = Gen
Charge = -8
Spin = Singlet
E(UB3LYP) = -4376.76502390 a.u.
RMS Gradient Norm = 0.00004987 a.u.
Imaginary Freq = 0
Dipole Moment = 9.9134 Debye
Optimization completed Atomic coordinates

Mo	0.16875400	1.55908700	-1.46033600
Mo	-2.34660400	-0.28121500	-0.52163400
Mo	0.51989100	-1.69945900	-1.12543900
O	-3.27480400	0.87209900	-1.92295600
O	1.46116100	-1.47800300	-2.75505400
O	-0.72668600	-2.98178100	-2.04561400
O	-3.02987200	-1.75102300	-1.65244300
O	-1.02950200	-1.41868500	0.13635700
O	1.22758400	1.19097100	-3.03197500
O	-1.18265000	2.37319000	-2.64977000
N	1.55631000	-2.74656000	-0.20213900
N	-3.55493200	-0.26372800	0.71551500

O	-0.81215700	-0.29731600	-2.25366000
O	-1.30825900	1.27294400	-0.15506000
N	1.15945600	0.06227600	-0.48016900
N	1.00608600	2.91207300	-0.70543600
C	3.22218700	-4.46832700	-0.18105700
H	3.06182900	-4.57945100	-1.09034200
C	2.66821100	-3.38809300	1.89697800
C	2.48587400	-3.53059600	0.52449100
C	3.68940400	4.87170200	0.84369500
H	4.60083400	5.03581400	0.75857100
C	1.68320600	3.80983200	0.09018200
C	2.94799900	5.56551600	1.82371900
H	3.38033100	6.15822000	2.39540700
C	0.97914300	4.50974400	1.07235800
H	0.05976200	4.39098700	1.14677200
C	3.90507600	3.15818600	-0.97037800
H	4.47102800	3.75800200	-1.46196000
H	3.33182000	2.68744700	-1.57988900
H	4.44727600	2.52838300	-0.48985600
C	3.07337500	3.93693400	-0.00558900
C	4.30123200	-5.05306600	1.78607300
H	4.89305500	-5.60305900	2.24667600
C	1.62437800	5.37701600	1.93751300
H	1.14290000	5.82665800	2.59392400
C	3.60899900	-4.11944700	2.48308200
H	3.79552500	-3.98904100	3.38476100
C	-4.60459300	0.05507100	2.86212800
H	-3.75412700	0.13439600	3.22999300
C	-5.75337300	0.19274900	3.68824200
H	-5.67228400	0.35220700	4.60080800
C	1.86044300	-2.54324200	2.67794000
H	2.34143100	-1.73716400	2.87948200
H	1.62699500	-2.98939300	3.49525900
H	1.06121500	-2.32606200	2.19241300
C	-4.75560000	-0.20020800	1.49349900
C	4.01314000	-0.37203400	-0.51022600
H	3.82790600	-1.30888900	-0.60846200
H	4.96066900	-0.23976700	-0.43137200
H	3.68688600	0.10101200	-1.27929200
C	-6.16241700	-0.68623400	-0.53838300
H	-5.61466100	-0.09487700	-1.05987300
H	-7.07879400	-0.59656300	-0.81009600
H	-5.87531200	-1.59210300	-0.67482500
C	-6.99382800	0.08184300	3.09078800
H	-7.76866200	0.15493800	3.59992500
C	-7.07190100	-0.13930700	1.72473700
H	-7.91601300	-0.15551700	1.33490500
C	-6.03238300	-0.32754300	0.94475800
C	1.34320200	0.86885400	1.90422300
H	0.43297100	1.05974600	1.89742900
C	1.98752900	0.34203600	0.72364300
C	4.05306700	0.49540000	1.85464100
H	4.98149700	0.47904600	1.80348700
C	2.07693300	1.08117500	3.01864400
H	1.65574400	1.37366100	3.79444900
C	4.16660200	-5.22502500	0.41778700
H	4.69422700	-5.82455500	-0.05883300

C	3.34543700	0.13557900	0.69691900
C	3.51600900	0.85982300	3.02541000
H	4.02915700	0.96495300	3.79384400

Mulliken charges with hydrogens summed into heavy atoms:

1	Mo	0.514321
2	Mo	0.493156
3	Mo	0.540614
4	O	-0.395379
5	O	-0.402844
6	O	-0.419235
7	O	-0.389875
8	O	-0.370484
9	O	-0.417120
10	O	-0.416669
11	N	-0.431475
12	N	-0.436095
13	O	-0.332916
14	O	-0.372907
15	N	-0.423139
16	N	-0.447871
17	C	-0.198862
19	C	-0.054699
20	C	0.011290
21	C	-0.305832
23	C	0.017408
24	C	-0.294086
26	C	-0.220200
28	C	-0.126378
32	C	-0.093382
33	C	-0.300160
35	C	-0.253658
37	C	-0.230954
39	C	-0.198356
41	C	-0.286120
43	C	-0.085957
47	C	0.004787
48	C	-0.070311
52	C	-0.138599
56	C	-0.319245
58	C	-0.261577
60	C	-0.084186
61	C	-0.025675
63	C	0.066934
64	C	-0.157521
66	C	-0.147062
68	C	-0.278033
70	C	-0.021028
71	C	-0.240622

Sum of Mulliken charges with hydrogens summed into heavy atoms = -8.00000

$\{Mo_6O_{14}[NC_6H_4(o\text{-}CH_3)]_4[\mu_2\text{-}NC_6H_4(o\text{-}CH_3)]\}^{2-}$
b3lyp/gen opt freq pop=full int=grid=ultrafine
C N O H 0
6-31+G(d)
****
Mo 0
LANL2DZ

****
Calculation Type = FREQ
Calculation Method = UB3LYP
Basis Set = Gen
Charge = -8
Spin = Singlet
E(UB3LYP) = -6627.63296130 a.u.
RMS Gradient Norm = 0.00004561 a.u.
Imaginary Freq = 0
Dipole Moment = 5.0201 Debye
Optimization completed Atomic coordinates

Mo	0.18062100	0.43242600	-1.64093700
Mo	-0.90225800	-2.69628400	-1.63236800
Mo	-2.47640400	-0.32057200	0.08188300
Mo	1.85287400	-1.81607500	-0.02459600
Mo	-0.82179700	-2.66436300	1.73365000
Mo	0.26145700	0.47361400	1.65229000
O	-2.51533200	-1.83610500	-1.28084800
O	1.92109800	-0.38508800	1.33781000
O	-1.07093400	-3.62429300	0.08045800
O	-0.33250300	-1.11783700	2.72910100
O	-1.19011700	-3.84604900	2.92491900
O	-2.48284900	-1.82176700	1.36729800
O	-1.71596400	0.77118400	1.38240200
O	-1.29699400	-3.88601900	-2.76614000
O	1.90223500	-0.39297500	-1.35556100
O	1.03836400	-2.93323500	-1.31921000
O	-0.33406300	-1.16807800	-2.67886500
O	1.05583900	-2.90388500	1.36536500
N	0.64489400	1.65543600	2.86857100
N	-4.15407200	0.09905200	0.09748400
O	-0.26131900	-0.98861800	0.03927300
O	-1.76536500	0.73053800	-1.33851800
N	0.48948900	1.58001600	0.02348500
N	0.51407700	1.68094200	-2.83742700
N	3.51712600	-2.36502700	-0.07789700
C	2.02906400	2.31436200	4.70975600
H	2.36874300	1.44864100	4.71263400
C	0.48025200	3.92232600	3.81105900
C	1.04409700	2.64988800	3.79512100
C	5.29091500	-4.01490000	0.00850100
H	4.69010300	-4.68016700	0.25604000
C	4.84200300	-2.70454600	-0.19024500
C	5.33195200	-0.29355400	-0.85162000
H	4.54900400	-0.28177500	-1.40702900
H	6.03485900	0.20146000	-1.27889000
H	5.12844800	0.10696600	-0.00324800
C	2.00894500	4.63791300	-4.40645300
H	2.83128800	5.06072300	-4.50578600
C	5.76872600	-1.69158000	-0.64484900
C	0.68395200	2.82140300	-3.59105900
C	0.87234200	5.17087400	-5.05111700
H	0.94760300	5.95537900	-5.54500500
C	-0.42192900	3.37636900	-4.23858600
H	-1.24482700	2.94628600	-4.18596300
C	3.12309500	2.99478500	-2.87926200
H	3.87057200	2.95157600	-3.48021500

H	2.94936400	2.12101800	-2.52139300
H	3.32394400	3.60026800	-2.16191600
C	1.91875900	3.47903600	-3.61687100
C	1.90651900	4.46010400	5.57960400
H	2.16108200	5.08492000	6.21968400
C	-0.31609900	4.55503500	-4.95728700
H	-1.06472400	4.91789300	-5.37294000
C	6.63449100	-4.32112500	-0.16411500
H	6.94336800	-5.18647600	-0.02044200
C	0.96348900	4.81077500	4.67165000
H	0.65032200	5.68617200	4.65093900
C	-6.17049700	1.40841900	-0.07846200
H	-5.63903200	2.17112800	-0.05144700
C	-7.58007100	1.52745000	-0.22005800
H	-7.98829700	2.36106500	-0.27679500
C	-0.60632100	4.27234900	2.99068800
H	-0.29250500	4.79726500	2.25061200
H	-1.23997200	4.78335600	3.49952700
H	-1.02856700	3.47545800	2.66153600
C	-5.58347500	0.14083600	0.02029100
C	2.92781500	2.98017000	0.67707400
H	2.80970600	2.67908900	1.58098700
H	3.69434100	3.55607900	0.62923000
H	3.06023000	2.22273700	0.10223800
C	-5.71963500	-2.36915900	0.17930500
H	-4.97204100	-2.44818000	-0.41781400
H	-6.35550100	-3.06289800	-0.01047600
H	-5.41537300	-2.45316900	1.08595400
C	-8.32319700	0.36418200	-0.27055000
H	-9.24894400	0.40130100	-0.35145600
C	7.06482200	-2.03465700	-0.77507800
H	7.67383800	-1.37692700	-1.02291900
C	7.52640700	-3.29735700	-0.56180600
H	8.42939700	-3.48738700	-0.67757300
C	-7.67503100	-0.85890500	-0.19958400
H	-8.18873900	-1.62865400	-0.29095300
C	-6.38330900	-1.00229500	-0.01172300
C	-0.59604000	3.79425200	-0.51357900
H	-1.36450700	3.34062600	-0.77566800
C	0.56544700	3.06321400	-0.06303200
C	1.73095100	5.11114300	0.06624800
H	2.54759000	5.54798900	0.15071200
C	-0.55427800	5.14417900	-0.55001100
H	-1.31633900	5.61873800	-0.79266100
C	2.50837000	3.21215700	5.59708700
H	3.19990000	3.00561100	6.18370100
C	1.73223600	3.71900500	0.24647800
C	0.66379400	5.86847200	-0.21581800
H	0.69819600	6.79767200	-0.19944900

Mulliken charges with hydrogens summed into heavy atoms:

1	Mo	0.805824
2	Mo	0.713209
3	Mo	0.776344
4	Mo	0.783967
5	Mo	0.723282
6	Mo	0.805033
7	O	-0.315822

8	O	-0.307600
9	O	-0.328073
10	O	-0.314988
11	O	-0.296114
12	O	-0.319419
13	O	-0.302271
14	O	-0.274712
15	O	-0.307990
16	O	-0.313995
17	O	-0.314835
18	O	-0.325012
19	N	-0.356753
20	N	-0.365793
21	O	-0.384969
22	O	-0.310898
23	N	-0.406842
24	N	-0.365583
25	N	-0.368012
26	C	-0.019788
28	C	0.021239
29	C	0.076446
30	C	-0.039703
32	C	0.079364
33	C	0.003486
37	C	-0.044317
39	C	0.018952
40	C	0.075374
41	C	-0.056551
43	C	-0.018773
45	C	0.006612
49	C	0.024335
50	C	-0.049442
52	C	-0.039531
54	C	-0.043496
56	C	-0.045945
58	C	-0.027885
60	C	-0.039248
62	C	0.013838
66	C	0.076925
67	C	0.004392
71	C	0.013315
75	C	-0.058176
77	C	-0.057798
79	C	-0.067249
81	C	-0.050080
83	C	0.013713
84	C	-0.011669
86	C	0.086351
87	C	-0.045730
89	C	-0.033291
91	C	-0.039132
93	C	0.005702
94	C	-0.060218

Sum of Mulliken charges with hydrogens summed into heavy atoms = -2.00000.

9. References

1. Proust, A., Thouvenot, R., Chaussade, M., Robert, F., Gouzerh, P. Phenylimido derivatives of $[Mo_6O_{19}]^{2-}$: syntheses, X-ray structures, vibrational, electrochemical, 95Mo and 14N NMR studies. *Inorg. Chim. Acta.* **224**, 81-95 (1994).
2. Gouzerh, P., Proust, A. Main-Group Element, Organic, and Organometallic Derivatives of Polyoxometalates. *Chem. Rev. (Washington, D C)* **98**, 77-111 (1998).
3. Strong, J. B. *et al.* A New Class of Functionalized Polyoxometalates: Synthetic, Structural, Spectroscopic, and Electrochemical Studies of Organoimido Derivatives of $[Mo_6O_{19}]^{2-}$. *J. Am. Chem. Soc.* **122**, 639-649 (2000).
4. Strong, J. B., Haggerty, B. S. Rheingold A. L., Maatta E. A. A superoctahedral complex derived from a polyoxometalate: the hexakis(arylimido)hexamolybdate anion $[Mo_6(NAr)_6O_{13}H]$. *Chem. Commun. (Cambridge)*, 1137-1138 (1997).
5. Strong, J. B., Ostrander, R., Rheingold, A. L., Maatta, E. A. Ensheathing a Polyoxometalate: Convenient Systematic Introduction of Organoimido Ligands at Terminal Oxo Sites in $[Mo_6O_{19}]^{2-}$. *J. Am. Chem. Soc.* **116**, 3601-3602 (1994).
6. Clegg, W. *et al.* Functionalization of $[Mo_6O_{19}]^{2-}$ with aromatic amines: synthesis and structure of a hexamolybdate building block with linear difunctionality. *J. Chem. Soc., Chem. Commun.* 455-456 (1995).
